# Hsf1 and Hsp90 orchestrate temperature-dependent global transcriptional remodelling and chromatin architecture in *Candida albicans*

**DOI:** 10.1038/ncomms11704

**Published:** 2016-05-26

**Authors:** Michelle D. Leach, Rhys A. Farrer, Kaeling Tan, Zhengqiang Miao, Louise A. Walker, Christina A. Cuomo, Robert T. Wheeler, Alistair J. P. Brown, Koon Ho Wong, Leah E. Cowen

**Affiliations:** 1 Aberdeen Fungal Group, University of Aberdeen, Institute of Medical Sciences, Foresterhill, Aberdeen AB25 2ZD, UK; 2 Department of Molecular Genetics, University of Toronto, Toronto, Ontario, Canada M5S 1A8; 3 Genome Sequencing and Analysis Program, Broad Institute of MIT and Harvard, Cambridge, Massachusetts 02142, USA; 4 Faculty of Health Sciences, University of Macau, Macau SAR 999078, China; 5 Department of Molecular and Biomedical Sciences, University of Maine, Orono, Maine 04469, USA

## Abstract

Fever is a universal response to infection, and opportunistic pathogens such as *Candida albicans* have evolved complex circuitry to sense and respond to heat. Here we harness RNA-seq and ChIP-seq to discover that the heat shock transcription factor, Hsf1, binds distinct motifs in nucleosome-depleted promoter regions to regulate heat shock genes and genes involved in virulence in *C. albicans*. Consequently, heat shock increases *C. albicans* host cell adhesion, damage and virulence. Hsf1 activation depends upon the molecular chaperone Hsp90 under basal and heat shock conditions, but the effects are opposite and in part controlled at the level of Hsf1 expression and DNA binding. Finally, we demonstrate that Hsp90 regulates global transcription programs by modulating nucleosome levels at promoters of stress-responsive genes. Thus, we describe a mechanism by which *C. albicans* responds to temperature via Hsf1 and Hsp90 to orchestrate gene expression and chromatin architecture, thereby enabling thermal adaptation and virulence.

## Abstract (web summary)

The transcription factor Hsf1 and the molecular chaperone Hsp90 modulate the heat shock response in the pathogen *Candida albicans*. Here, Leach *et al*. reveal a complex interplay between the two factors that regulates the expression of genes involved in the heat shock response and virulence.

## Graphical abstract

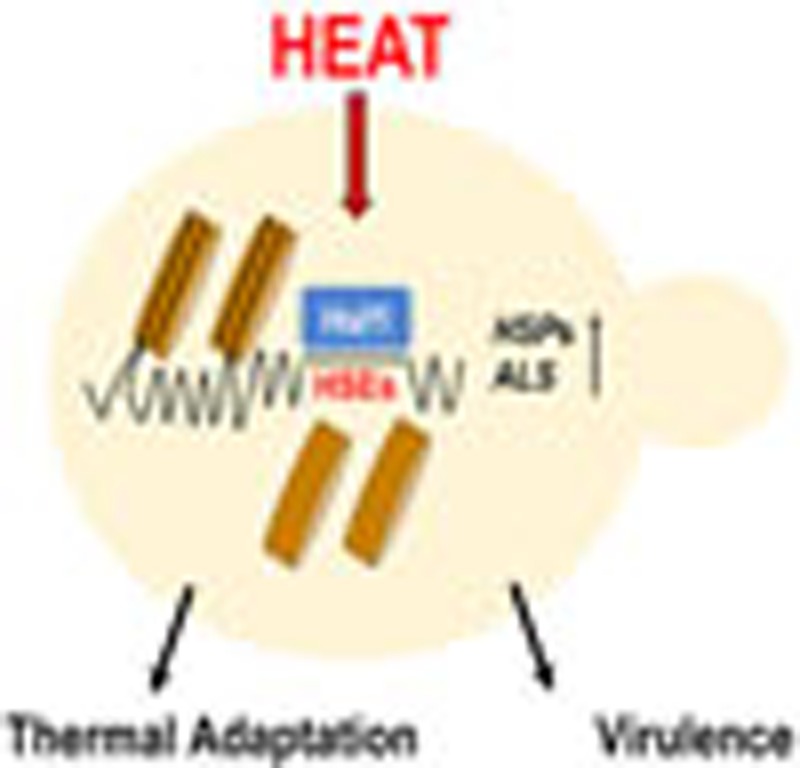

Compared with the environmental extremes encountered by saprobes in nature, mammalian hosts provide relatively stable niches for commensal microorganisms and pathogens. Nevertheless, microorganisms that colonize mammals are continually challenged with a myriad of environmental stimuli such as temperature fluctuations, osmotic imbalances, oxidative and weak-acid stresses, as well as nutrient limitation^1^. Survival, colonization and infection depend upon activation of environmental response pathways that have been fine-tuned over evolutionary time to drive physiological adaptation^2^,^3^.

The potential for optimization of circuitry governing sensing and responding to host conditions is greatest for microbes that are natural members of commensal microbiomes. This includes the diverse bacteria that promote host immune function and health, as well as fungi that have only recently been appreciated to play important roles in defining commensal communities^4^. Commensal fungi are capable of becoming opportunistic pathogens that can cause life-threatening infections. One such fungus is *Candida albicans*, which has evolved as a relatively harmless commensal of the mucous membranes and digestive tracts of healthy individuals. When host antimicrobial defences are compromised, this yeast can cause superficial mucosal infections in otherwise healthy individuals^5^, and up to 400,000 life-threatening systemic infections in immunocompromised patients per year^6^.

With fever as a ubiquitous host response to infection, it follows suit that the capacity to sense and respond to thermal cues is one of the most important and conserved stress responses in nature. The heat shock response is characterized by global pausing of translation elongation^7^ and remodelling of gene expression programs distinguished by the induction of heat shock proteins (HSPs), such as chaperones that aid in protein folding^3^. The master regulator of this transcriptional response is the heat shock transcription factor (HSF), which is conserved from yeasts to humans^8^,^9^. Hsf1 is essential for viability in *C. albicans* and other yeasts, likely due to its function in enabling core gene expression programs^10^,^11^. HSF binds to the major groove of *cis*-acting DNA sequence motifs termed heat shock elements (HSE), characterized by tandem inverted repeats of the consensus sequence 5′-nGAAn-3′ (refs 12, 13). In *Saccharomyces cerevisiae*, transcription of ∼10% of genes is modulated in response to heat shock^3^, and nearly 3% of genes are directly bound by Hsf1 (ref. 14). Transcription initiation in response to elevated temperature is also influenced by nucleosome positioning, with heat shock resulting in increased nucleosome occupancy at repressed promoters and decreased occupancy at active promoters^15^.

Cellular adaptation to thermal stress is modulated by complex functional relationships between Hsf1 and the molecular chaperone Hsp90, which influence signalling and gene expression. As with many chaperones, Hsf1 enables basal expression and temperature-dependent activation of Hsp90-encoding genes^16^. Hsp90 controls the Hsf1-HSE regulon through an autoregulatory circuit^17^,^18^, activation of which is essential for virulence of *C. albicans*^19^. Beyond Hsf1, Hsp90 stabilizes diverse cellular regulators^20^, and exerts additional control on gene expression. In *Drosophila*, Hsp90 targets paused RNA polymerase II (ref. 21) and in *S. cerevisiae*, deletion of *HSC82* (the constitutively expressed *HSP90* isoform) delays nucleosome removal from the *GAL1* promoter^22^. Despite the profound impact of temperature-dependent regulation of Hsf1 and Hsp90 on virulence traits in fungal pathogens^23^,^24^,^25^, the mechanisms by which these global regulators orchestrate temperature-dependent signalling, gene expression and virulence has remained elusive. Here, we use genome-scale approaches to explore how *C. albicans* responds to thermal insults, revealing that Hsf1 and Hsp90 govern nucleosome positioning, gene expression and virulence traits.

## Results

### Hsf1 binds genes required for stress adaption and virulence

**Figure 1:**
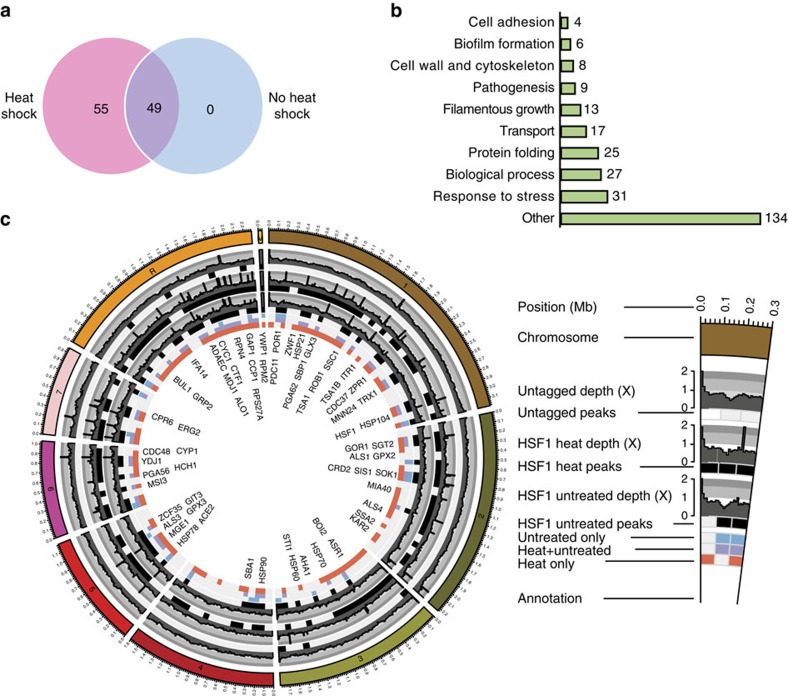
Hsf1 target genes. (**a**) Venn diagram depicting number of Hsf1 peaks in the absence (no heat shock, blue) or presence (heat shock, pink) of a 30–42 °C heat shock. (**b**) GO Slim Mapper analysis was performed on the ChIP-seq data set to identify the processes of all genes bound by Hsf1 in the absence and presence of a 30–42 °C heat shock. Number of Hsf1 target genes represented under each category are indicated. Other categories include, but are not limited to, response to drug, cell cycle, response to chemical, organelle localization, protein catabolic process, translation, ribosome biogenesis, signal transduction and cell development. (**c**) Circos plot illustrating peaks of genes bound by Hsf1 (systematic names) in the absence and presence of a 30–42 °C heat shock. Each circle from the periphery to the core represents the following: chromosomal location; normalized read depth of peaks from untagged samples, heat shocked samples and untreated samples; key of condition under which the peak is present (untreated, untreated with heat shock and heat shock only); and name of gene bound. All boxes represent ≥1 peak that falls within a 100 kb non-overlapping window.

Using chromatin immunoprecipitation (ChIP) of TAP-tagged Hsf1 (Hsf1-TAP) followed by sequencing (ChIP-seq), we set out to identify the genome-wide direct targets of Hsf1 in the absence (30 °C) or presence of heat shock (30–42 °C). Distinct Hsf1-TAP ChIP-seq signals were identified at 49 genomic sites in the absence of heat shock. Upon heat shock, 104 Hsf1-binding sites were identified, including all 49 observed in the absence of heat stress (Fig. 1 and Supplementary Data 1). We hereafter refer to the 49 sites as constitutive targets, and the remaining 55 sites as heat shock-dependent targets. The majority of binding events were found at promoter regions close to transcription start sites (Supplementary Fig. 1a), with transcription of genes closest to Hsf1-binding sites being highly upregulated upon heat shock (Supplementary Fig. 1b). Our results suggest that Hsf1 constitutively binds to a set of target promoters before heat shock pending activation.

Many Hsf1 target genes identified in our study encode proteins implicated in stress responses such as molecular chaperones, oxidative stress regulators, and ubiquitination and proteolysis factors. Unexpectedly, Hsf1 regulates its own expression and also bound upstream of genes required for adhesion, filamentous growth and pathogenesis (Fig. 1b,c and Supplementary Data 2). GO term analysis of Hsf1-bound genes revealed enrichment of the categories ‘protein-folding' (hypergeometric distribution *P*=1.87e−20) ‘response to heat' (hypergeometric distribution *P*=0.00074) and ‘entry into the host' (hypergeometric distribution *P*=0.0052) (Supplementary Data 3), suggesting that Hsf1 is not only required for regulating *HSPs* in response to heat shock but also genes involved in virulence.

Metagene analysis was performed to compare the regulation of Hsf1 target genes with constitutive and heat-induced binding at their promoters in response to heat shock. Transcription (RNA polymerase (Pol) II occupancy measured by Pol II ChIP-seq) of genes with constitutive Hsf1 binding increased dramatically in response to heat shock (Supplementary Fig. 1c). This is accompanied by a drastic increase in Hsf1-binding levels (Supplementary Fig. 1d), suggesting that Hsf1 is critical for their upregulation during thermal insults. In contrast, heat-induced Hsf1 targets display lower activation in response to heat shock (Supplementary Fig. 1c), with consistently lower levels of Hsf1 binding at these promoters (Supplementary Fig. 1d). Taken together, these observations suggest that the magnitude of Hsf1-dependent activation during heat shock is contingent upon its level of binding before heat shock.

*C. albicans* Hsf1 target genes may also be regulated by additional transcription co-factors. For example, *ALS3* is co-regulated by Nrg1 and Tup1 (ref. 26), the *ALS1* promoter is bound by Bcr1, Tec1, Efg1, Ndt80 and Brg1, and the *ROB1* promoter is bound by Tec1, Efg1, Ndt80 and Rob1 (ref. 27). Therefore, we tested whether upregulation of these Hsf1 targets in response to heat shock is contingent upon Hsf1. To achieve this, we used a *tetO-HSF1/hsf1*Δ strain to deplete *HSF1* and monitored the expression of genes required for thermal adaption (*HSP90*, *HSP104* and *HSP21*)^28^, adhesion and virulence (*ALS1* and *ALS3*)^29^,^30^ and biofilm formation (*ROB1*)^27^ (Supplementary Fig. 2). Depletion of *HSF1* abrogated the heat shock-dependent upregulation of key *HSPs*: *HSP90*, *HSP104* and *HSP21*. Further, we found that *ALS3*, *ALS1* and *ROB1* expression increased significantly in response to heat shock, and that depletion of *HSF1* reduced this upregulation (Supplementary Fig. 2). Thus, Hsf1 regulates the expression of not only heat shock genes but also key virulence genes in response to heat shock.

### Hsf1 binds canonical and non-canonical heat shock elements

**Figure 2:**
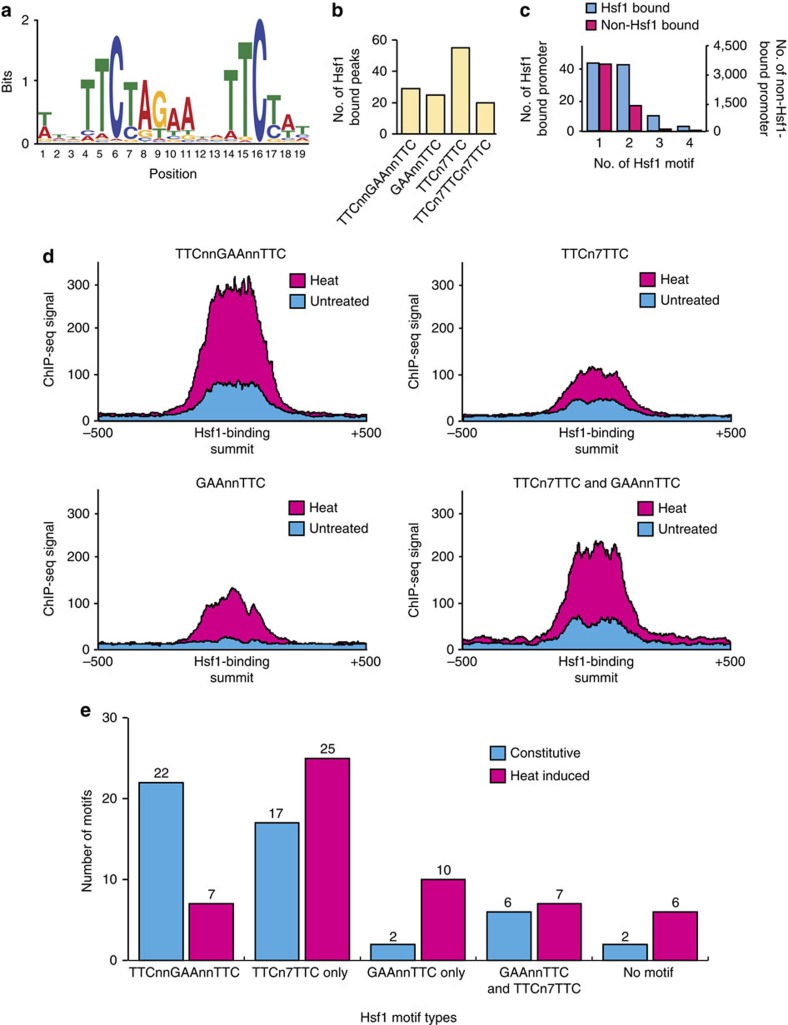
Hsf1-binding motifs. (**a**) *De novo* motif analysis using MEME on Hsf1-binding sites identified this highly significant sequence (*E*-value=1.5e−164). (**b**) Bar graph representing the number of Hsf1 targets containing one of four motifs identified using *de novo* motif discovery and manual inspection. (**c**) Histogram illustrating the number of different Hsf1 motifs found in Hsf1-bound (blue) versus non-Hsf1-bound promoters (red). (**d**) Hsf1 binding signal as measured by Hsf1 ChIP-seq at promoters containing the indicated Hsf1 motifs under untreated (blue) and heat shock (red) conditions. (**e**) Bar graph illustrating number of Hsf1-binding events with the indicated motifs. Constitutively bound Hsf1 genes are represented in blue, heat induced Hsf1 genes are represented in red.

The findings that there are two classes of Hsf1-binding targets (constitutive and heat-induced), and that Hsf1 activation level is correlated with its binding pattern prompted us to search for enriched motif(s) at the promoters of Hsf1 targets. We began with *de novo* motif discovery on the full set of Hsf1-binding sites (200 bp spanning the summit of 104 Hsf1-TAP ChIP-seq peaks) and identified one highly significant consensus sequence (*E*-value=1.5e−164) (Fig. 2a). This motif, conforming to a pattern of three inverted nGAAn repeats, is similar to both the human^31^ and *S. cerevisiae*^14^ Hsf1 recognition motifs, suggesting that *C. albicans* Hsf1 probably functions as a trimeric complex as with other HSF homologues^8^. However, only 29 out of the 104 Hsf1 ChIP-seq peaks contained the consensus sequence TTCnnGAAnnTTC (Fig. 2b), indicating that Hsf1 likely recognises additional motif(s) in *C. albicans*.

We extended our analysis to search for additional sequence element(s). Using *de novo* motif discovery and manual inspection, we found that the motifs GAAnnTTC and TTCn_7_TTC (25 and 55 out of 104, respectively) are also enriched among Hsf1-bound regions (Fig. 2b). The three motifs identified are found within 100 bp of the centre of Hsf1-TAP ChIP-seq peaks with strong ChIP-seq signals (Supplementary Fig. 3a and Supplementary Data 4), indicating they are probable Hsf1-binding elements. The latter motif is a shorter version of the step-type non-canonical HSE (nTTCn_7_TTCn_7_TTC) identified in *S. cerevisiae*^32^, while the former motif has not been reported for Hsf1 in other organisms. It is the most predominant motif among the three, present at a higher frequency among Hsf1-bound peaks than the *S. cerevisiae* step-type non-canonical motif (Fig. 2b). These findings suggest that *C. albicans* and *S. cerevisiae* Hsf1 have related yet distinct binding affinities, and that *C. albicans* Hsf1 is capable of binding to nGAAn elements arranged in at least three configurations, presumably in different dimeric and trimeric forms.

A further 4,879 genes with the Hsf1 consensus sequences present in their promoter were identified, but no detectable Hsf1 binding was observed under our ChIP-seq experimental conditions (Fig. 2c and Supplementary Data 4). These genes were enriched for functions related to transport, stress responses, filamentous growth, cell cycle, translation and metabolism, and may be bound by Hsf1 in response to specific cues. Our analysis also highlighted that the TTCnnGAAnnTTC and TTCn7TTCn7TTC motifs are highly enriched among Hsf1-bound promoters compared with promoters that have Hsf1 motifs without Hsf1 binding (Supplementary Fig. 3b). Moreover, the majority of Hsf1-bound promoters contain at least one type of Hsf1 motif (Supplementary Fig. 3c). We also discovered that many Hsf1 targets contain multiple Hsf1 motifs (for example, GAAnnTTC repeats 1 kb upstream of the ATG), with two promoters (*TSA1B* and *HSP70*) having as many as nine motifs (Supplementary Fig. 3c), suggesting a strong selection pressure for Hsf1 regulation of these target genes.

Hsf1 binds to the three identified motifs with different binding affinities. The TTCnnGAAnnTTC element is most strongly bound, while GAAnnTTC and TTCn7TTC have a reduced but still significant binding affinity (Fig. 2d). The TTCnnGAAnnTTC element was enriched among promoters with constitutive Hsf1 binding, whereas heat-induced Hsf1-binding events were associated with the other two motifs with weaker Hsf1-binding affinity (Fig. 2e). Consistently, the level of Hsf1 bound at heat-induced promoters was lower than the level bound at constitutive promoters in both the absence and presence of heat shock (Supplementary Fig. 1d). Together, our results suggest that the differential binding affinity of Hsf1 in the absence and presence of heat is associated with the configuration of nGAAn elements at target promoters.

### Nucleosome positioning regulates Hsf1 binding to DNA

**Figure 3:**
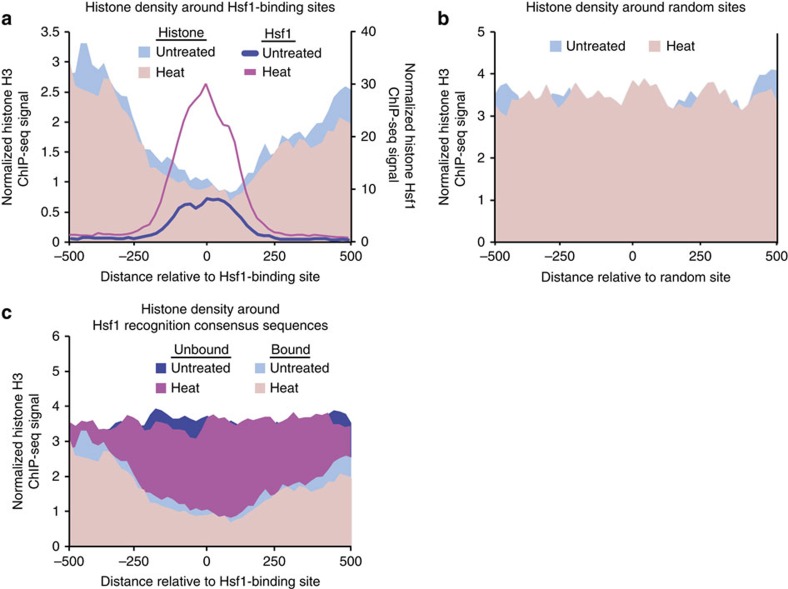
Nucleosome positioning affects Hsf1 DNA binding. (**a**,**b**) Schematic plots illustrating nucleosome density (shaded area) and Hsf1 binding (lines) at (**a**) Hsf1-binding sites and (**b**) random genomic regions before (blue) and after (red) heat shock. (**c**) Nucleosome density around genomic regions containing Hsf1 recognition consensus sequences with (bound) or without (not bound) Hsf1 (as determined by Hsf1-TAP ChIP-seq analysis) before (blue) and after (red) heat shock. Nucleosome and Hsf1-TAP levels were measured by Histone H3 and Hsf1-TAP ChIP-seq, respectively.

Nucleosomes have a profound impact on the accessibility of DNA to transcription factors and on transcription initiation^15^, thus we sought to determine if nucleosomes affect Hsf1 DNA binding. At genomic regions with Hsf1 binding, nucleosomes (measured by histone H3 ChIP-seq) were depleted near Hsf1 motifs (Fig. 3a). As a control, we examined nucleosome density at 104 random genomic regions, finding that nucleosome depletion is specific to Hsf1-bound regions (Fig. 3b). Further, we analysed genomic regions containing an Hsf1 consensus sequence that were not bound by Hsf1 in our experimental conditions, and found that nucleosome density was consistently high across these recognition motifs (Fig. 3c). Together, this suggests that nucleosome positioning at Hsf1 motifs influences the ability of Hsf1 to recognize and bind DNA.

This result prompted us to investigate whether the different binding affinities at the three Hsf1 binding motifs were also mediated through nucleosome density, but no obvious correlation was found (Supplementary Fig. 4a). Specifically, nucleosome levels at both Hsf1-binding sites (Fig. 3a) and Hsf1-regulated promoters (1 kb upstream of ATG) (Supplementary Fig. 4b) did not significantly differ upon heat shock, suggesting that Hsf1 activation and heat shock do not invoke significant nucleosome eviction at these heat shock-induced promoters.

### Heat shock increases virulence of *C. albicans in vivo*

Our data demonstrate that Hsf1 binds to genes involved in virulence, adhesion and biofilms during heat shock, but these genes may play an as yet undetermined role in thermal adaptation. To assess this, *als3*Δ/*als3*Δ and *rob1*Δ*/rob1*Δ mutants, along with their wild-type counterparts, were heat shocked and cell viability was determined by colony forming units (CFUs). The mutants displayed no significant heat shock sensitivity compared with their respective wild-type controls (Supplementary Fig. 5a). Thus, these genes are not required for thermal adaptation.

**Figure 4:**
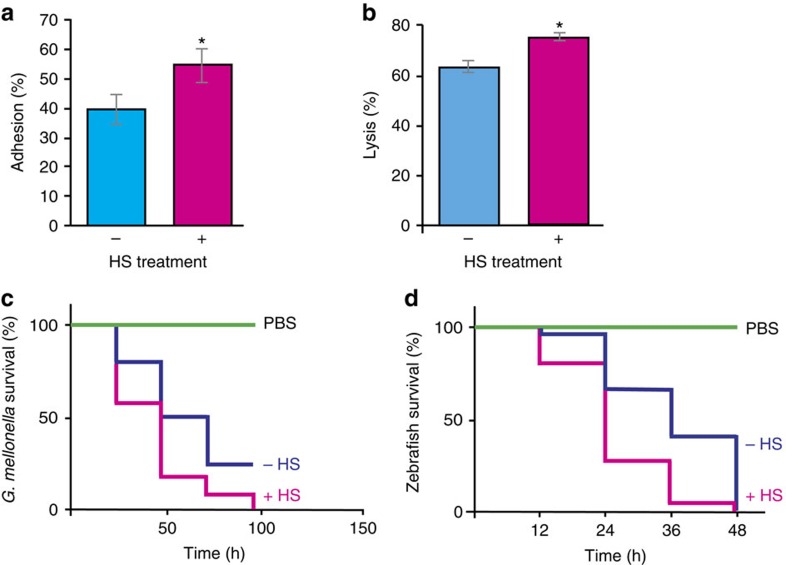
Heat shock increases pathogenicity traits. (**a**) Increased adhesion of *C. albicans* wild-type (SN95) cells to TR146 epithelial cells post 30–42 °C HS. Results represent percentage of adhered cells from 30 °C grown cells (no HS) or 30–42 °C heat-shocked cells (HS) compared with control CFUs. Data represent mean values ± s.e.m. from two independent biological replicates. Student *t*-test, *P<0.05 compared with wild type. (**b**) Increased cell damage by *C. albicans* wild-type (SN95) cells post 30–42 °C HS to TR146 epithelial cells. Culture medium was assessed for LDH release as a measure of epithelial cell damage of TR146 cells after incubation with *C. albicans* grown at 30 °C (no HS) or subjected to a 15 min 30–42 °C HS. Data represent mean values ± s.e.m. from two independent biological replicates. Student t-test, *P<0.05 compared with the wild type. (**c**) HS increases virulence in the *G. mellonella* model of infection. Twenty larvae per treatment group were injected with 2.5 × 10^5^ cells of *C. albicans* wild-type (SN95) cells grown at 30 °C (no HS) or subjected to a 15 min 30–42 °C HS. PBS was used as a control. Data are representative of four biological replicates. Significance was determined using Log-rank (Mantel–Cox) test, with a *P*<0.001 (**d**) HS increases *C. albicans* virulence in the zebrafish model of infection. Ninety zebrafish larvae per treatment group were injected with 15–20 *C. albicans* wild-type (SN95) cells grown at 30 °C (no HS), wild-type cells subjected to a 15 min 30–42 °C HS or with a PBS control. Significance was determined using Log-rank (Mantel–Cox) test, with a *P*<0.0001. HS, heat shock.

*C. albicans* has evolved in endothermic animals as an opportunistic pathogen. Thus we postulated that it exploits thermal insults to upregulate its virulence program via Hsf1. Further, our previous studies established that a prior heat shock protects cells from oxidative stress, which could enable *C. albicans* to resist killing by the host immune response^18^. To test the impact of heat shock on virulence traits, we utilized the TR146 oral epithelial carcinoma cell lines as a well-established model to investigate adhesion and cell damage^33^,^34^. Cells primed with heat shock displayed 15% greater adherence than cells grown at 30 °C alone (Student's *t*-test *P*<0.05) (Fig. 4a). This is likely due to the Hsf1-dependent upregulation of *ALS* genes, which are effectors of epithelial adhesion and invasion^35^,^36^. Next, we assayed epithelial cell damage by measuring the release of lactate dehydrogenase (LDH) from TR146 cells. After 20 h co-incubation, damage increased when *C. albicans* had received a prior heat shock (*P*<0.05) (Fig. 4b). These results cannot be attributed to morphological changes during heat shock, as cells remain in the yeast form up to 120 min post 30–42 °C heat shock^18^. Our data suggest that a drastic heat shock activates a *C. albicans* program associated with increased host cell adhesion and damage.

To evaluate the effect of heat shock on virulence, we utilized two complementary infection models, the greater wax moth (*Galleria mellonella*) and zebrafish (*Danio rerio*). *G. mellonella* larvae infected with heat shocked *C. albicans* exhibited increased mortality rates compared with controls (*P*<0.05, Log-rank test) (Fig. 4c). These results were recapitulated with the zebrafish infection model, with increased mortality of zebrafish infected with *C. albicans* cells that had received a prior heat shock compared with controls (*P*<0.05, Log-rank test) (Fig. 4d). Thus, an acute heat shock increases virulence in two models of *Candida* infection.

We further corroborated our results using a temperature shift that more closely recapitulates physiological conditions associated with a febrile host. *C. albicans* has been isolated from skin, which is typically 33 °C; it can then become internalized, and subsequently local inflammation or fever can raise temperatures as high as 41 °C. We previously established that Hsf1 initiates the classic heat shock response during slow thermal transitions that would mimic fever, but also during sharp transitions from 37–42 °C (ref. 37). To determine if different temperature shifts have comparable effects on virulence traits, we repeated the TR146 adhesion assay using a 37–42 °C heat shock, and found that adhesion increased in cells that had received a prior heat shock, albeit not to the same extent as cells that received a 30–42 °C heat shock (Supplementary Fig. 5b). Further, the impact of Hsf1 on expression of key genes for heat shock (*HSP90*, *HSP104* and *HSP21*) and virulence (*ALS1*, *ALS3, ACE2* and *ROB1*) was also maintained in response to a 37–42 °C heat shock, though not to the same magnitude as with the 30–42 °C heat shock. (Supplementary Fig. 5c). Therefore, our data suggest that Hsf1 coordinates cellular responses to host-relevant thermal shifts.

### Hsp90 remodels global transcription during heat shock

The emergent paradigm from decades of research has been that Hsp90 physically interacts with Hsf1 to repress its activating function, such that when Hsp90 functional capacity is overwhelmed by elevated temperature or other perturbations, the repression is relieved and Hsf1 activates the heat shock response^18^. However, recent studies have implicated Hsp90 in directly regulating gene expression through chromatin remodelling, RNA polymerase II pausing and nucleosome removal at target promoters^21^,^22^,^38^. To assess the impact of Hsp90 on the transcriptional response to heat shock, we performed RNA-seq in a wild-type and *tetO-HSP90/hsp90Δ* strain at 10, 30 and 60 min post heat shock. In wild-type cells, heat shock elicited rapid, large-scale remodelling of gene expression. At 10 min post heat shock, 766 genes were >2-fold upregulated, increasing to 1,119 genes by 60 min, representing ∼18% of *C. albicans* genome (Supplementary Fig. 6a,b and Supplementary Data 5). GO term analysis across the three time points supported the hypothesis that a primary effect of heat shock is protein unfolding (Supplementary Fig. 6c), as well as modulation of genes involved in the proteasome/ubiquitination and oxidative stress response. Notably, *C. albicans* also upregulated genes related to cell cycle, biofilm formation and pathogenesis, in keeping with our findings that an acute heat shock increases pathogenesis (Fig. 4). Heat shock also downregulated a large portion of protein-encoding genes in *C. albicans*, enriched in different metabolic processes and ribosome biogenesis (Supplementary Fig. 6a).

**Figure 5:**
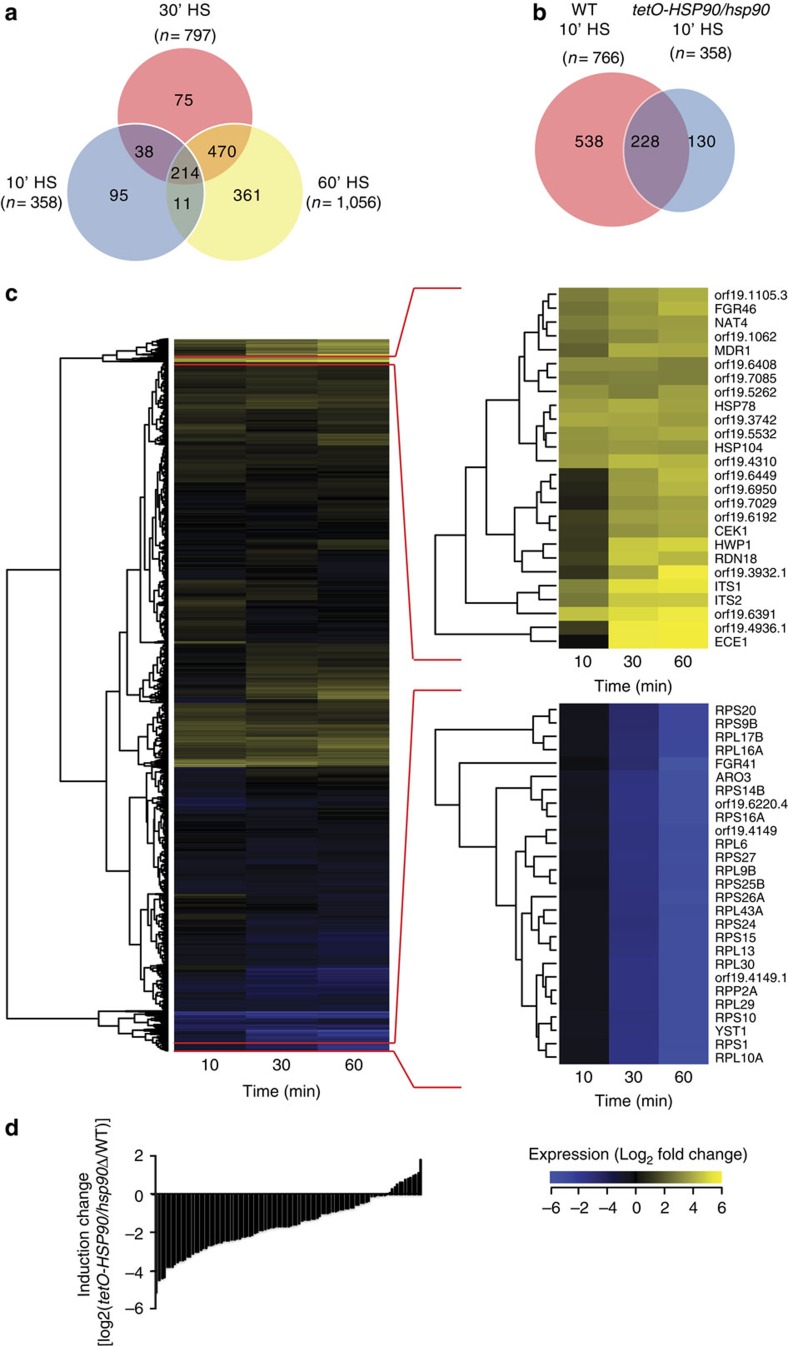
Depletion of Hsp90 changes the genome-wide heat shock signature. (**a**) Venn diagram depicting number of upregulated genes 10, 30 and 60 min post 30–42 °C HS in *HSP90*-depleted cells. Overlap between samples is illustrated, with the total number of genes upregulated at each time point represented under each time point. (**b**) Venn diagram illustrating gene expression overlap between wild-type and *tetO-HSP90/hsp90Δ* strains 10 min post 30–42 °C HS. (**c**) Expression pattern of the *tetO-HSP90/hsp90Δ* strain grown in the presence of 20 μg ml^−1^ doxycycline for transcriptional repression of *HSP90* compared with the same strain 10, 30 and 60 min post 30–42 °C heat shock. Yellow represents greater than Log_2_-fold increase in gene expression, blue represents a Log_2_-fold or greater decrease in gene expression. Columns represent individual time points. (**d**) Histogram illustrating the effect of *HSP90* depletion on heat-induced expression changes of Hsf1 target genes. Transcription induction (increase in PolII level) of Hsf1-target genes upon heat shock in the absence of *HSP90* was compared with that in wild-type, and the differences (induction change) are expressed in log_2_ scale. HS, heat shock.

Hsp90 had a profound impact on the transcriptional response to heat shock. Upon *HSP90* depletion, 10 min post-heat shock, only 358 of 6,197 genes were >2-fold upregulated, compared with 766 in wild-type cells, and of these, only 228 were common to the wild-type program (Fig. 5a,b). These genes were enriched for protein folding and refolding, similar to wild-type cells (Supplementary Table 1). Advancing to 30 and 60 min post treatment, the number of genes upregulated >2-fold increased to 1,056. However, these genes were enriched for cytoskeleton organization, nuclear division, chromosome segregation and cell cycle-related processes, reinforcing functional connections between Hsp90 and cell cycle control at high temperature in *S. cerevisiae*^39^. By 60 min, an enrichment of ERAD (endoplasmic-reticulum-associated protein degradation) pathway genes were found, while protein-folding categories were no longer enriched, suggesting that the combined stress is so pernicious that cellular resources are diverted to targeting unfolded proteins for degradation. Notably, depleting *HSP90* in the absence of any stress caused upregulation of 515 genes by >2-fold (Supplementary Data 5). GO term analysis reveals an enrichment of genes involved in metabolic processes (Supplementary Data 6), but no enrichment of genes involved in general stress responses. This suggests the transcriptional effects observed in response to *HSP90* depletion are not due to non-specific stress from mis-folded proteins. Metabolic and ribosome biogenesis genes remained downregulated upon depletion of *HSP90* during heat shock (Fig. 5c). Therefore, Hsp90 is required for normal upregulation of most heat shock inducible genes during heat shock.

A strong temporal signature underlies the impact of *HSP90* depletion. Expression of over 75% of Hsf1 target genes was drastically affected upon *HSP90* depletion after a 10 min heat shock (Fig. 5d), and the observed effect is mainly due to the loss of transcriptional induction upon heat shock (Supplementary Fig. 7a). However, changes in expression of Hsf1 targets between wild-type and *HSP90*-depleted cells gradually became less pronounced post heat shock (Supplementary Fig. 7b). Thus, Hsp90 is crucial for rapid mobilization of the heat shock response.

### Hsp90 modulates Hsf1 DNA binding and expression

**Figure 6:**
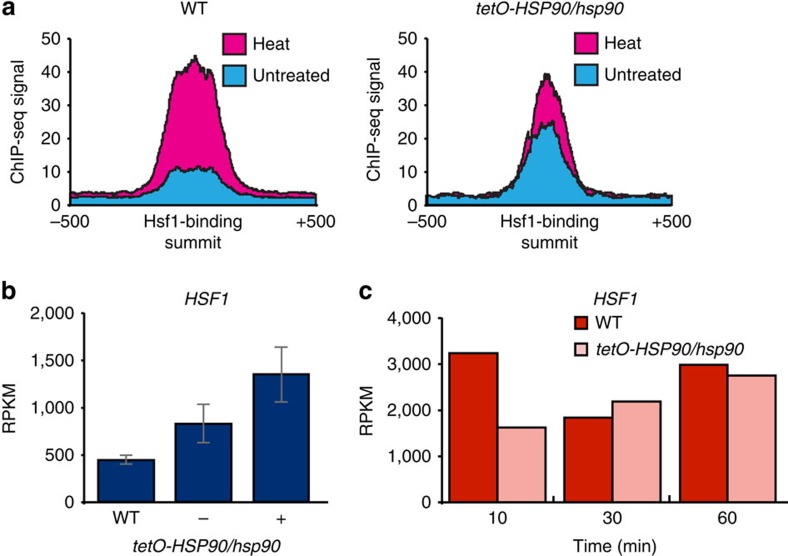
Hsp90 modulates expression of *HSF1*. (**a**) Depletion of *HSP90* increases the binding signal of Hsf1 in the absence of heat shock compared with the wild type. Blue represents untreated, red represents heat shocked cells. (**b**) Expression of *HSF1*, measured by RNA-seq, increases upon *HSP90* depletion in the absence of heat shock. *HSF1* expression in wild-type (SN95, WT) or *tetO-HSP90/hsp90*Δ strain in the absence (−) or presence (+) of 20 μg ml^−1^ doxycycline to repress *HSP90* expression. Data represent mean values ± s.d. from three independent biological replicates (**c**) Heat induction of *HSF1* expression in wild-type (dark red) versus *tetO-HSP90/hsp90Δ* (light red) cells over a 10, 30 or 60 min 30–42 °C heat shock.

Hsp90 is known to interact with Hsf1 in *C. albicans*, inhibiting its full activation^18^. We therefore expected Hsf1 binding to increase upon *HSP90* depletion. Indeed, overall Hsf1-TAP ChIP-seq signals at Hsf1 targets increased upon *HSP90* depletion compared with wild-type (Fig. 6a). Moreover, 18% of Hsf1 targets were upregulated >1.5-fold upon *HSP90* depletion in the absence of heat shock, supporting the established model that Hsp90 represses Hsf1 function in the absence of heat shock. These genes were enriched for GO categories including response to heat, protein unfolding and heat acclimation (Supplementary Table 2). In contrast, Hsf1 binding at target promoters 15 min post heat shock was markedly reduced upon *HSP90* depletion (Fig. 6a), which would account for the reduced expression of Hsf1 target genes during early heat shock.

Before heat shock, *HSF1* expression is elevated upon *HSP90* depletion (Fig. 6b). This is consistent with the increase in Hsf1 DNA binding and Hsf1-dependent gene expression we observed. Strikingly, the induction of *HSF1* expression in response to heat shock observed in wild-type cells was abolished 10 min post heat shock upon depletion of *HSP90* (Fig. 6c). Thus, under basal conditions, Hsp90 represses Hsf1 such that depletion of *HSP90* induces *HSF1* expression, binding and induction of genes required for protein re-folding. However, Hsp90 is required for the rapid mobilization of the heat shock response, such that depletion of *HSP90* impairs induction of *HSF1* leading to a delayed response.

### Hsp90 maintains nucleosome-free regions at promoters

**Figure 7:**
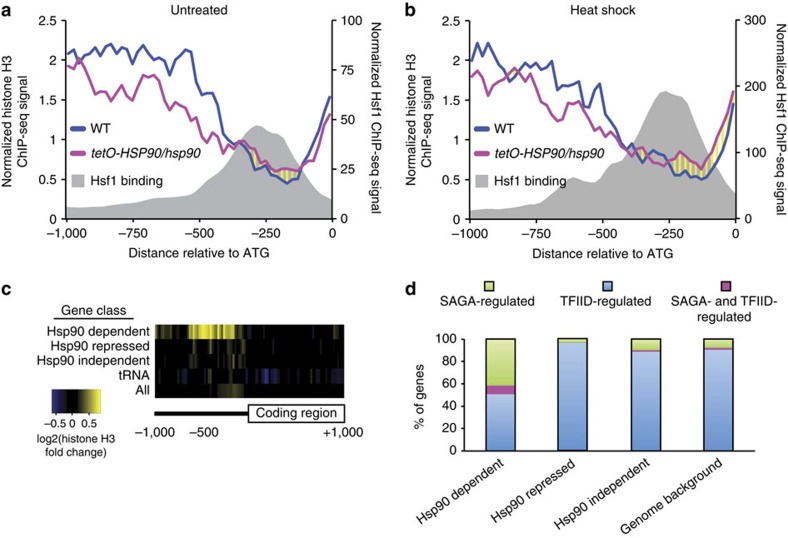
Hsp90 is required for maintaining nucleosome-free regions. Schematic diagrams showing nucleosome density in wild-type (SN95, blue) and *tetO-HSP90/hsp90* (red) cells, as well as Hsf1-TAP-binding position (grey shade) at Hsf1 regulated promoters with (**a**) and without (**b**) heat shock. (**c**) Heat map illustrating nucleosome density changes upon *HSP90* depletion 1 kb upstream and downstream of indicated classes of genes. Yellow represents an increase and blue represents a decrease in nucleosome density. (**d**) Histogram representing the per cent of Hsp90-dependent or—repressed genes regulated by SAGA or TFIID compared with Hsp90-independent genes and the genome background.

Given the striking differences in global gene expression upon heat shock in wild-type versus *HSP90*-depleted cells, and the discovery that nucleosome positioning modulates Hsf1 binding to DNA, we postulated that Hsp90 may affect gene expression via nucleosome positioning in *C. albicans*. Using histone H3 ChIP-seq analysis of wild-type and *tetO-HSP90/hsp90*Δ, we found that *HSP90* depletion caused nucleosome re-arrangements at Hsf1-regulated promoters under both untreated and heat shock conditions (Fig. 7a,b). Specifically, we observed that nucleosome density at distal promoter regions was reduced. However, nucleosome density increased at the nucleosome-depleted region of Hsf1-regulated genes that overlap with Hsf1-binding sites (Fig. 7a,b). At the level of individual promoters, 71 and 87% of Hsf1 regulated promoters showed an increase in histone H3 levels at the nucleosome-free region upon *HSP90* depletion before and after heat shock, respectively (Supplementary Fig. 7c,d). Importantly, the increased histone H3 levels correlated with the effect of *HSP90* depletion on heat induction (as measured in Fig. 5c) (Supplementary Fig. 7e). Taken together, these findings suggest that the effect of Hsp90 on Hsf1 DNA binding could also be mitigated through alterations of the chromatin context at promoters by masking Hsf1 DNA-binding motifs.

To assess whether the impact of Hsp90 on chromatin is Hsf1-specific or more global, we extended the analysis to genes with increased (Hsp90 Repressed), decreased (Hsp90 Dependent) and unchanged (Hsp90 Independent) expression as measured by PolII ChIP-seq upon *HSP90* depletion, as well as to the RNA polymerase II transcribed tRNA genes as a control (Supplementary Data 7). We identified significant effects on chromatin upon *HSP90* depletion only among the category of genes with decreased expression (Fig. 7c). Strikingly, nucleosome positioning was observed primarily at the nucleosome-depleted region but not the distal promoter and gene body of Hsp90-dependent genes (Fig. 7c and Supplementary Fig. 7f). Nucleosome-depleted regions are a critical property of core promoters for transcription factor function and transcription initiation^40^, suggesting that Hsp90 orchestrates gene expression in *C. albicans* via nucleosome positioning.

Hsp90 may modulate nucleosome positioning by multiple mechanisms. First, one of the 143 genes that are Hsp90-repressed is an ortholog of *S. cerevisiae FPR3*, orf19.1030. This gene is involved in nucleosome assembly and deposition^41^. Second, the transcription factor, Cbf1, which is involved in nucleosome positioning in *S. cerevisiae*^42^, was repressed upon *HSP90* depletion. Third, 8 out of 11 genes known to encode histones are repressed when *HSP90* is depleted. These genes were also repressed upon heat shock, indicating that Hsp90 is required for full expression of histone genes, and sequestration of Hsp90 during heat shock impairs histone gene expression. Fourth, by identifying orthologs of *S. cerevisiae* genes controlled by SAGA (Spt-Ada-Gcn5-acetyltransferase) or TFIID (transcription factor II D), we discovered that Hsp90-repressed genes are almost exclusively controlled by TFIID, whereas Hsp90-dependent genes are dominated by SAGA (Fig. 7d); this is in keeping with the role of SAGA in regulating stress-induced genes^43^. In both *S. cerevisiae* and *C. albicans*, Hsp90 genetically interacts with *SPT3*, a component of the SAGA complex^38^,^44^. SAGA acetylates promoter nucleosomes, which facilitates displacement of nucleosomes^45^; this suggests that nucleosome density may increase at Hsp90 dependent genes due to impaired function of the SAGA complex upon Hsp90 compromise. These mechanisms are not mutually exclusive, and may all contribute to the impact of Hsp90 on transcriptional responses to cellular stress.

## Discussion

The capacity to coordinate sensing and responding to temperature is a driving force for microbial evolution. Beyond enabling adaptation to thermal stress, growth at physiological temperatures is a fundamental requirement for pathogenesis, and mammalian endothermy may have evolved to restrict fungal infections^46^. Our goal was to determine if temperature modulates virulence in a pathogen that has evolved in a relatively stable thermal environment. Using RNA-seq and ChIP-seq, we observed that heat shock induces profound transcriptional remodelling in *C. albicans*, with upregulation of ∼18% of the genome, which is controlled in large part by Hsf1 in concert with Hsp90. Many of the genes induced by heat shock in *C. albicans* encode HSPs required to re-fold thermally denatured proteins, and components of the ubiquitination/proteolysis pathway that target unfolded proteins for degradation. We also observed that heat shock induces genes involved in filamentation, pathogenesis and adhesion, which were found to be directly regulated by Hsf1 during heat shock (Fig. 1b). This includes the *ALS* family of genes that are required for adhesion^29^,^30^, and *ROB1*, a key regulator of biofilm formation^27^. We found that the transcriptional program induced by a 30–42 °C heat shock in *C. albicans* is associated with increased host cell adherence, host cell damage, and virulence (Fig. 4). These findings were validated with a more physiologically relevant heat shock of 37–42 °C; although the magnitude of effects on virulence traits was smaller, it is likely that additional stresses encountered in the human host would amplify the impact of temperature stress. Together, our findings support a model in which Hsf1 has evolved as a key temperature sensor, primed to both protect the cell from thermal insults and enable exploitation of a compromised host.

Motif analysis of Hsf1 targets demonstrates that most heat-induced Hsf1 targets, including *ALS1*, *ALS3* and *ALS4*, contain the motifs GAAnnTTC and TTCn_7_TTC, whereas many constitutively bound Hsf1 targets, including *HSF1*, contain the motif TTCnnGAAnnTTC (Fig. 2e). This suggests that Hsf1 may regulate expression of virulence genes via a distinct mechanism from its regulation of heat shock genes. Contrary to studies in *S. cerevisiae*^47^, we found that histone binding levels do not change significantly in response to heat shock (Fig. 3c). This suggests three things: first, Hsf1-binding sites are primed for binding; second, upregulation of *HSF1* during heat shock drives the increase in Hsf1 binding; and third, Hsf1 activation does not involve histone eviction. Further, histone levels remain similar at different Hsf1 motifs (Supplementary Fig. 4a,b), suggesting that Hsf1-binding sites (and consequently target genes) are pre-determined by chromatin architecture. Regulation at this level suggests that Hsf1 is able to provide robustness that promotes survival in diverse hostile host niches and rapid cellular responses to signals from the host immune system or microbiota to initiate transitions from commensal to pathogenic states.

The capacity of Hsf1 to orchestrate gene expression programs and cellular adaptation is modulated by complex functional relationships with Hsp90. Under basal conditions, depletion of *HSP90* leads to the upregulation of a subset of Hsf1 targets (19 out of 104): those enriched for protein folding and response to heat (Supplementary Table 2). This likely occurs through an increase in *HSF1* expression and Hsf1 binding to DNA upon *HSP90* depletion (Fig. 6a,b). This fits with the model that Hsp90 represses Hsf1 function^48^,^49^,^50^, and our previous observations that depletion of *HSP90* induces Hsf1 phosphorylation and upregulates Hsf1 targets^18^. In response to heat shock, depletion of *HSP90* impairs the rapid global transcriptional response, which could contribute to the reduced virulence of strains depleted of *HSP90* in a murine model of candidiasis^23^. The delay may be due in part to the decrease in *HSF1* induction resulting in a reduced Hsf1-binding signal during heat shock upon *HSP90* depletion (Fig. 6a,c). These effects are likely exacerbated by the increase in promoter nucleosome density observed upon *HSP90* depletion at Hsf1 target genes and genes with Hsp90-dependent expression (Fig. 7). This is consistent with findings that Hsp90 enables nucleosome removal from the *S. cerevisiae GAL1* promoter and recruitment of transcriptional machinery^22^, and suggests a broader role for Hsp90 in nucleosome architecture.

Such alterations in nucleosome architecture likely occur through multiple mechanisms. First, Hsp90 modulates expression of numerous genes that influence nucleosome biology, including the chaperone *FPR3*, transcription factor *CBF1* and histone genes. Second, Hsp90 may influence function of the SAGA complex via interactions with Spt3 in *C. albicans*^44^. Notably, in *S. cerevisiae* Hsp90 interacts with multiple components of the SAGA complex, including Ada2, Gcn5, Spt3, Spt4 and Spt15 (ref. 38). The histone acetyltransferase activity of the SAGA complex facilitates promoter nucleosome displacement^45^. Third, given that *S. cerevisiae* Hsp90 interacts with the chromatin remodelling factors Rvb1/2, Vps1, Swr1 and Aor1 (ref. 38), Hsp90 may modulate chromatin states through interaction with chromatin remodelling factors. Fourth, Hsp90 may regulate nucleosome eviction. Consistent with this possibility, Hsp90 associates with histone H3.1 and the histone chaperone t-NASP in mammals^51^, with depletion of Hsp90 increasing the levels of soluble H3 and H4 (ref. 52). The resultant increase in nucleosome occupancy that could arise by these mechanisms would inhibit transcription^15^, with depletion of *HSP90* reducing expression of over 75% of Hsf1 target genes after a 10 min heat shock. Thus, Hsp90 modulates global transcriptional responses to stress via nucleosome occupancy, and protein homoeostasis via repressive effects on Hsf1.

The mechanisms through which core eukaryotic cellular regulators modulate host-pathogen interactions are only beginning to be understood. *C. albicans* has evolved elaborate mechanisms to thrive in diverse and hostile environments, where it must resist host defences and mitigate polymicrobial interactions in the highly competitive communities that flourish in commensal and pathogenic states. The coordinated control of the heat shock response with a transcriptional program that promotes pathogenesis suggests that *C. albicans*, as well as other similar pathogens have adapted to febrile temperatures as a ubiquitous host response to infection, exploiting the temperature shift, along with other host responses, as a cue to activate virulence programs. This provides a stunning example of thermal fluctuation as a driving force for host-pathogen co-evolution in the fungal kingdom.

## Methods

### Strains and growth conditions

All strains are listed in Supplementary Table 3. Strains were grown in YPD (1% yeast extract, 2% bactopeptone, 2% glucose)^53^. To impose an instant heat shock of 30–42 °C, cells were first grown in YPD at 30 °C to exponential phase, and then half of the volume was removed and mixed with an equal volume of medium that had been pre-warmed to 54 °C in flasks that had been pre-warmed at 42 °C. To impose an instant heat shock of 37–42 °C, cells were grown in YPD at 37 °C to exponential phase, and then half of the volume was removed and mixed with an equal volume of medium that had been pre-warmed to 47 °C in flasks that had been pre-warmed at 42 °C. Cells were left at 30 or 37 °C (untreated controls) or grown at 42 °C (heat shock) for the times indicated. Doxycycline (BD Biosciences) was added to YPD medium at a concentration of 20 μg ml^−1^ to mediate transcriptional repression from the *tetO* promoter.

### RNA-seq

To comprehensively study the heat shock response, we performed high-throughput sequencing of cDNA (RNA-seq) made from RNA isolated from *C. albicans*. *C*. *albicans* wild-type (CaLC239, SN95) and *tetO-HSP90*/*hsp90Δ* (CaLC1441) cells were grown overnight at 30 °C in YPD. Stationary phase cultures were split, adjusted to an OD_600_ of 0.05 in YPD with or without 20 μg ml^−1^ doxycycline (BD Biosciences) and grown for 6 h to ensure that *HSP90* is depleted, but cells are still viable^18^. Cells were then subjected to a heat shock for 10, 30 and 60 min, as described in growth conditions. Cells were harvested at 3,000 r.p.m. for 2 min at 4 °C, washed once with 1 × PBS and frozen rapidly in liquid nitrogen. RNA was extracted with Triazol (GibcoBRL: Grand Island, NY), as described previously^54^. RNA integrity was assayed on an Agilent Bioanalyser 2,100 (Stockport, UK). TruSeq RNA-seq libraries were prepared according to the manufacturer's instructions (Illumina, Cambridge, UK), and sequenced on the Illumina HiSeq2000 platform. Over 80% of raw sequences aligned to the *C. albicans* SC5314 genome sequence (*Candida Genome Database*)^55^. Gene expression analysis was performed using Partek Genomics Suite software, version 6.6; 2015. Approximately 20 million reads were generated per sample, over 80% of which mapped to *C. albicans* ORFs. DESeq^56^ was used to normalize the data sets and calculate the fold changes and their statistical significance on the basis of three independent biological replicates for each condition (Supplementary Data 5). GOSeq was then used to examine the enrichment of GO terms^57^.

### ChIP-seq

*C*. *albicans* untagged wild-type (CaLC239, SN95) and Hsf1-TAP-tagged (CaLC2993) cells were grown to mid-log phase (OD_600_ 0.6) at 30 °C (untreated) and subjected to a 15 min heat shock as described in growth conditions. Cells were poured into 50 ml falcon tubes containing 1.2 ml of 37% formaldehyde up to the 40 ml mark and incubated with gentle rocking for 20 min at room temperature, after which 10 ml of 2.5 M glycine was added to stop the crosslinking and cells were mixed for 2 min. Cells were harvested at 3,000 r.p.m. for 2 min at 4 °C, washed twice with ice-cold 1 × TBS and frozen rapidly in liquid nitrogen. Chromatin was extracted as described previously^58^. Briefly, cells were lysed in 500 μl of FA lysis buffer (150 mM NaCl) for 3 min using a Bullet Blender. Lysis was repeated three times with a 3 min incubation on ice in between. Chromatin was pelleted by centrifugation at 14,000 r.p.m. for 15 min at 4 °C and subsequently re-suspended in 500 μl of FA lysis buffer (150 mM NaCl). Sonication was used to shear chromatin DNA to around 100–300 bp in size. Chromatin solution was recovered by centrifugation at 14,000 r.p.m. for 30 min at 4 °C and kept at −80 °C until use. Immuno-precipitation of TAP-tagged Hsf1 was performed by mixing 50 μl of chromatin extract with 450 μl of FA lysis (150 mM NaCl) and ∼15 μl packed IgG Sepharose 6 Fast Flow matrix (GE Healthcare) for 3 h at room temperature on an end-to-end rotator. Similarly, immuno-precipitation of histone H3 and PolII was executed by first mixing 50 μl of chromatin extract with 450 μl of FA lysis (150 mM NaCl) and 2 μg of anti-H3 (Abcam ab1791) or 2 μl of anti-PolII antibodies (Covance 8WG16), respectively, for 1.5 h, followed by incubating the mixture with ∼15 μl packed Protein A Sepharose (GE Healthcare) for another 1.5 h at room temperature on an end-to-end rotator. Subsequently, IgG or Protein A Sepharose matrix was transferred to a Corning Costar SpinX centrifuge tube filter and washed according to Fan and colleagues^58^. Immuno-precipitated chromatin DNA was de-crosslinked at 65 °C overnight and was purified using a QIAGEN PCR clean up purification column. Library preparation and multiplex Illumina Sequencing was carried out as described previously^59^,^60^. Libraries were sequenced by the Illumina HiSeq2500 platform. The nuclear and mitochondrial genome sequences and General Feature File for *C. albicans* isolate SC5314 v.A21-s02-m08-r01 were downloaded from www.candidagenome.org (GenBank project accession number PRJNA10701). Illumina reads were aligned to the genome using Bowtie with this parameter: −5 10 −k 1, --best^61^.

### Identification of Hsf1 targets

Binding sites of Hsf1-TAP were identified using MACS^62^. MACS-called peaks with a *P* values <0.05 were manually inspected and dubious binding sites (peaks with low signal in both untreated and heat conditions and/or portraying a strange shape) were removed from further analysis. Mapping the genes by their ATG position located closest to confident Hsf1-TAP ChIP-seq peaks, identifying direct Hsf1 target genes. A total of 103 genes were identified from 104 MACS peaks from data sets of untreated and heat shock conditions (Supplementary Data 1).

### *De novo* motif discovery

Sequences (200 bp) spanning the summit of the Hsf1-TAP ChIP-seq peaks were subjected to *de novo* motif analysis using MEME, DREME and HOMER. For extended analysis in identifying additional motifs, peaks with a canonical Hsf1-binding site (GAAnnTTC) 100 bp each side of the summit were removed and the same *de novo* motif analysis was carried out on the remaining peaks (Supplementary Data 4).

### Hsf1 binding and nucleosome density (histone H3) analysis

Hsf1 and histone H3 ChIP-seq signals were calculated by counting the number of sequencing read fragments that overlapped within 20 bp sliding windows across a 1 kb region spanning the summit of each peak or across the 1 kb promoter and the first 1 kb coding region of each target gene. The read count was then normalized to the total number of mapped reads in the respective data set. A median was taken for each 20 bp window, and the median (multiplied by a factor of 1,000,000 for *y* axis scaling for plotting purposes) was used in the ChIP-seq signal plot for the analysed peaks, promoter and coding regions (−1,000 to +1,000 with respect to ATG). Nucleosome free regions were systematically identified using the wild-type untreated histone H3 ChIP-seq data by scanning for the lowest Histone H3 signals across 20 bp windows within 500 bp upstream of ATG. If the identified window had zero histone H3 signal, the first 20 bp neighbouring window with a positive signal is set as the boundary of the nucleosome free region. Histone H3 signals (normalized to total number of mapped reads) at the identified regions were measured and compared between different samples and expressed as log_2_ fold change. To rule out that the observed effect of *HSP90* depletion (the increased H3 levels) is not due to a technical artefact, comparative analysis was performed between biological replicates. Two-sample Kolmogorov–Smirnov test was performed between untreated and heat shocked samples (Supplementary Fig. 7d; *P* values=7.723e−07 and 5.791e−09 for untreated and heat shock comparisons, respectively).

### Pol II ChIP-seq analysis

ChIP-seq against RNA polymerase II (Pol II) was used to determine active transcription activities. Transcription level was measured by the number of ChIP-seq fragments (sequencing reads) across the entire open reading frame of a gene normalized first to the length of the gene followed by the total number of mapped reads of a data set. The normalized number, which is similar to RPKM in the RNA-seq analysis, was used in expression comparisons between untreated and heat shock conditions as well as between wild-type and *tetO-HSP90/hsp90*Δ strains.

### Classification of Hsp90 targets

Gene expression (measured by PolII ChIP-seq) before and after heat shock in strains with or without *HSP90* depletion was compared. Genes with lower and higher expression upon *HSP90* depletion were classified as Hsp90-Dependent (fold change>-2) and Hsp90-Repressed (fold change>2), respectively, while genes whose expression remain unchanged (fold change between ±0.2 to 0) are referred to as Hsp90-Independent.

### qRT-PCR

To monitor gene expression changes in response to heat shock, strains SN95 (CaLC2993, wild-type) and CaLC2998 (*tetO*-*HSF1/hsf1*Δ) were grown overnight at 30 °C in YPD. Stationary phase cultures were split, adjusted to an OD_600_ of 0.2 in 10 ml YPD with or without doxycycline (BD Biosciences) and cultured at 30 °C for 16 h. This was necessary to ensure depletion of Hsf1. Cells were diluted once again to an OD_600_ of 0.2 in the same treatment conditions as the 16 h and were grown at 30 °C until mid-log phase (4 h). Cells were subjected to a 15 min 30–42 °C heat shock as described earlier and 50 ml was harvested from each culture at the specified time, centrifuged at 3,000 r.p.m. for 2 min at 4 °C, and washed once with 1 × PBS before snap freezing in liquid nitrogen. Pellets were stored at −80 °C before extraction. To monitor gene expression upon a 37–42 °C heat shock, wild-type cells (SN95, CalC239) were grown to mid-log phase in YPD at 37 °C before heat shock as described earlier. RNA was isolated using the QIAGEN RNeasy kit and cDNA synthesis was performed using the AffinityScript cDNA synthesis kit (Stratagene). PCR was carried out using the SYBR Green JumpStart Taq ReadyMix (Sigma-Aldrich) with the following cycle conditions: 95 °C for 3 min, 95 °C for 10 s and 60 °C for 30 s for 39 rounds, 95 °C for 10 s, 65 °C for 5 s. All reactions were done in triplicate using primers listed in Supplementary Table 4. Transcript levels were normalized to *ACT1*. Data were analysed using the BioRad CFX Manager software, version 3.1 (Biorad).

### Heat shock survival assays

To test heat shock survival in different mutants, *C. albicans rob1*Δ*/rob1*Δ (CaLC2739) and *als3*Δ*/als3*Δ (CaLC3115) mutants, along with their respective wild-type SN152 (CaLC2740) and BWP17 (CaLC3665) (Supplementary Table 3) were grown to mid-log phase in YPD at a starting OD_600_ of 0.1. Upon reaching mid-log phase, cells were left untreated or were stressed for one hour with a 30–42 °C heat shock, and CFUs determined by plating onto YPD plates. Viability was calculated as a percentage of survival after a heat shock compared with untreated cells. Experiment was replicated on three separate occasions.

### Culture of epithelial cells

The squamous carcinoma of buccal mucosa derived epithelial cell line TR146 was obtained as a gift from Julian Naglik, Kings College London. TR146 cells were routinely grown in DMEM medium containing 10% heat inactivated FBS (Gibco, 16,000,044) and × 1 antibiotic-antimycotic (Gibco, 15,240,062). When TR146 cells were grown in the presence of *C. albicans*, antibiotic-antimycotic was replaced with 1% penicillin-streptomycin (Sigma, P4333). Cells were maintained in a humidified incubator at 37 °C, 5% CO_2_. For adhesion, TR146 oral epithelial cell monolayers were grown to confluence in six-well tissue culture plates and serum-starved overnight. For cell damage assays 5 × 10^5^ cells were seeded in 24-well plates and cultured for 24 h before experiments.

### Cell adherence and damage assays

*C. albicans* wild-type (SN95, CaLC239) cells were grown to mid-log phase in YPD before subjection to a 15 min heat shock. To determine adhesion, untreated and heat shock treated cells were counted and 100 cells were added to replicate wells containing 1 ml serum-free DMEM for 90 min at 37 °C and 5% CO_2_ or plated onto replicate control plates. Non-adherent *C. albicans* cells were removed by aspiration, wells were washed twice with 1 ml PBS, and overlaid with molten Sabouraud's Dextrose Agar (SDA) at 40 °C (ref. 34). After 24 h at 30 °C the resulting colonies were counted and per cent adherence was calculated as (mean adherent CFU/mean total CFU) × 100. To determine cell damage, untreated and heat shock-treated wild-type (SN95, CaLC239) cells with a fungal:epithelial cell multiplicity of infection of 0.01 (ref. 34) were added to TR146 monolayers and incubated at 37 °C and 5% CO_2_ for 20 h. Control wells contained PBS alone. Following incubation, culture supernatant was collected and assayed for LDH using the Cytox 96 Non-Radioactive Cytotoxicity Assay kit (Promega) according to manufacturer's instructions. Replicate LDH measurements were made on the single well, and the assay was repeated on separate days. All data were analysed using a two-tailed t-test with *P*<0.05 considered significant.

### *Galleria mellonella* killing assay

Larvae in their final instar (Next Millennium Farms) were injected with *C. albicans* wild-type (SN95, CaLC239) cells grown at 30 °C or subject to a 15 min 30–42 °C heat shock. Briefly, overnight cultures were adjusted to an OD_600_ 0.1 in YPD. Cells were grown to mid-log phase before being subjected to the heat shock. *C. albicans* cells were washed with PBS and cell densities determined by haemocytometer counts. Dilutions were prepared in PBS and cells were injected at 2.5 × 10^5^ in 10 μl into the haemocoel via a distinct proleg, with 20 larvae per infection group. Kill curves were plotted and analysed by the Kaplan–Meier method (GraphPad Prism). Log-rank test was used to determine significance.

### Zebrafish care and maintenance

Zebrafish were maintained in re-circulating systems (Aquatic Habitats, Apopka, FL) at the University of Maine Zebrafish Facility with a 14/10 h light/dark cycle and water temperature at 28 °C. All zebrafish care and husbandry were performed as described previously^63^. All zebrafish care protocols and experiments were performed in accordance with NIH guidelines under Institutional Animal Care and Use Committee (IACUC) protocol A2012-11- 03.

Wild-type, AB, zebrafish larvae were maintained at a density of 150 per dish in 15 cm petri dishes containing 150 ml of E3 media (5 mM NaCl; 0.17 mM KCl; 0.33 mM CaCl_2_; 0.33 mM MgCl_2_; 2 mM HEPES; pH 6.8). E3 media was supplemented with 0.3 mg l^−1^ methylene blue for the initial 24 h to inhibit microbial growth. Larvae were cleaned by changing the E3 media daily. Zebrafish were raised in water containing 15 μg ml^−1^ of 1-phenyl-2-thiourea (PTU) (Sigma-Aldrich, St Louis, MO) to prevent pigmentation.

### Zebrafish hindbrain ventricle infection

*C. albicans* infection in the hindbrain ventricle was performed as previously described^64^. Briefly, Zebrafish at the prim25 stage (∼36 h post fertilization) were staged according to the method of Kimmel *et al*.^65^. Before infection larvae were manually dechorionated, and anaesthetized in Tris-buffered tricaine methane sulfonate (tricaine; 200 mg ml^−1^) (Western Chemicals, Inc., Frendale, WA). For each independent experiment, 30 larvae were microinjected with 4 nl of either PBS or 1 × 10^7^ cells per ml of *C. albicans* suspension through the otic vesicle into the hindbrain ventricle to achieve a dose of ∼15–20 yeast/larva. Larvae were then screened by microscopy immediately post-infection to ensure correct inoculum sizes and injection location. Data from three independent experiments were plotted. Kill curves were plotted and analysed by the Kaplan–Meier method (GraphPad Prism). Log-rank test was used to determine significance.

### Data availability

RNA-sequencing data sets are available at ArrayExpress (www.ebi.ac.uk) under accession code E-MTAB-4075. ChIP-seq data sets are available at the NCBI SRA database (http://www.ncbi.nlm.nih.gov) under accession code SRP071687. The authors declare that all other data supporting the findings of this study are available within the article and its supplementary information files, or from the corresponding author upon request.

## Additional information

**How to cite this article:** Leach, M. D. *et al*. Hsf1 and Hsp90 orchestrate temperature-dependent global transcriptional remodelling and chromatin architecture in *Candida albicans*. *Nat. Commun.* 7:11704 doi: 10.1038/ncomms11704 (2016).

## Supplementary Material

Supplementary Information Supplementary Figures 1-7, Supplementary Tables 1-4, Supplementary References

Supplementary Data 1 Constitutively bound and heat shock bound Hsf1 targets identified by ChIP-seq.

Supplementary Data 2 GO term processes of all Hsf1 bound genes. Hsf1 targets were catagorised into processes using Candida Genome Database GO Slim Mapper function.

Supplementary Data 3 GO terms of Hsf1 bound genes grouped by specific catagories using Candida Genome Database GO Term Finder function.

Supplementary Data 4 Hsf1 motif analysis. Bioinformatic analysis of C. albicans genome to identify the common Hsf1 motifs identified by ChIP-seq analysis in promoters (1 kb upstream of ATG) of all genes.

Supplementary Data 5 RNA-seq of wild-type and HSP90 depleted cells in the absence and presence of heat shock. Log2 Fold change between conditions tested and transcript levels of each sample for three biological replicates included.

Supplementary Data 6 GO terms of HSP90 depleted genes in the absence of any stress grouped by specific catagories using Candida Genome Database GO Term Finder function.

Supplementary Data 7 RNA polymerase II ChIP-seq in wild-type versus HSP90 depleted cells.
