# Supplementary material for: Hsf1 and Hsp90 orchestrate temperature-dependent global transcriptional remodelling and chromatin architecture in Candida albicans
Source: Nat Commun. 2016 May 26;7:11704. doi: 10.1038/ncomms11704 (PMC4894976; doi:10.1038/ncomms11704)
Supplement: Supplementary Information — Supplementary Figures 1-7, Supplementary Tables 1-4, Supplementary References [file ncomms11704-s1.pdf]

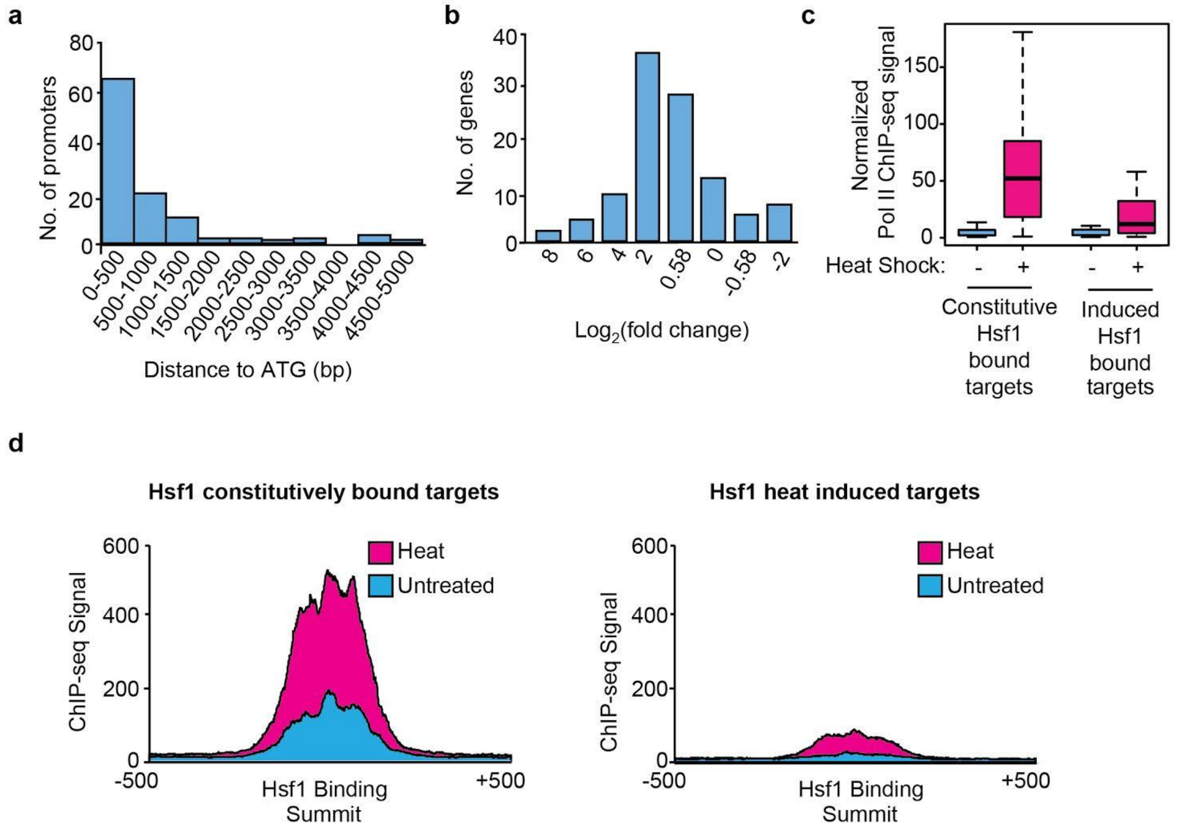

Supplementary Figure 1. Hsf1 displays differential binding to constitutive targets vs heat induced targets. (a) Binding sites of Hsf1, determined by ChIP-seq, were mapped to determine their distance from the start codon of the closest genes. Number of promoters for each distance are represented in this histogram. (b) Bar graph representing the number of Hsf1 targets with the indicated expression changes, as measured by RNA-seq (expressed as Log_2 [Fold Change] upon heat shock). (c) Box-plot depicting transcription (measured through polymerase II occupancy) of genes with constitutive (left) vs heat induced (right) Hsf1 binding at their promoters in the absence (blue) and presence (red) of a 15 minute 30˚C-42˚C heat shock. (d) Relative Hsf1 ChIP-seq signal of constitutively bound genes (left) versus heat induced genes (right), in the absence (blue) and presence (red) of heat shock.

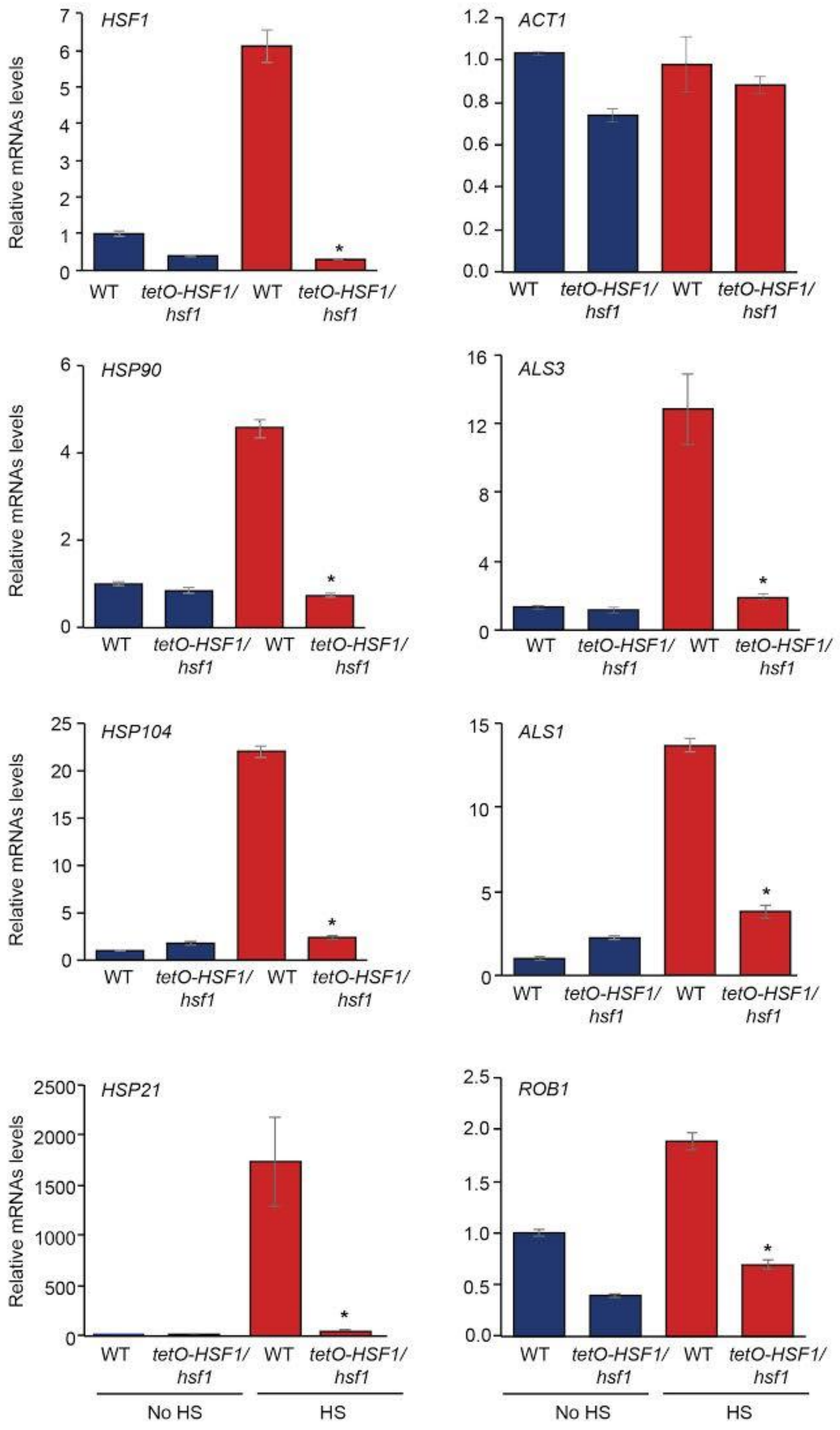

Supplementary Figure 2. Hsf1 is required for target gene expression in response to heat shock. *C. albicans* wild-type (WT) and *tetO-HSF1/hsf1*Δ cells were grown with 20 µg/ml doxycycline to exponential phase at 30˚C or subjected to a 15 minute 30˚C-42˚C heat shock (HS), and transcript levels of target genes were measured and normalised to the *ACT1* loading control. Unnormalised levels of *ACT1* are also displayed to illustrate that not all transcripts change in response to *HSF1* depletion. Data represent mean values +/- SD from two independent biological replicates. Student t-test, *p<0.05 compared to wild-type (WT).

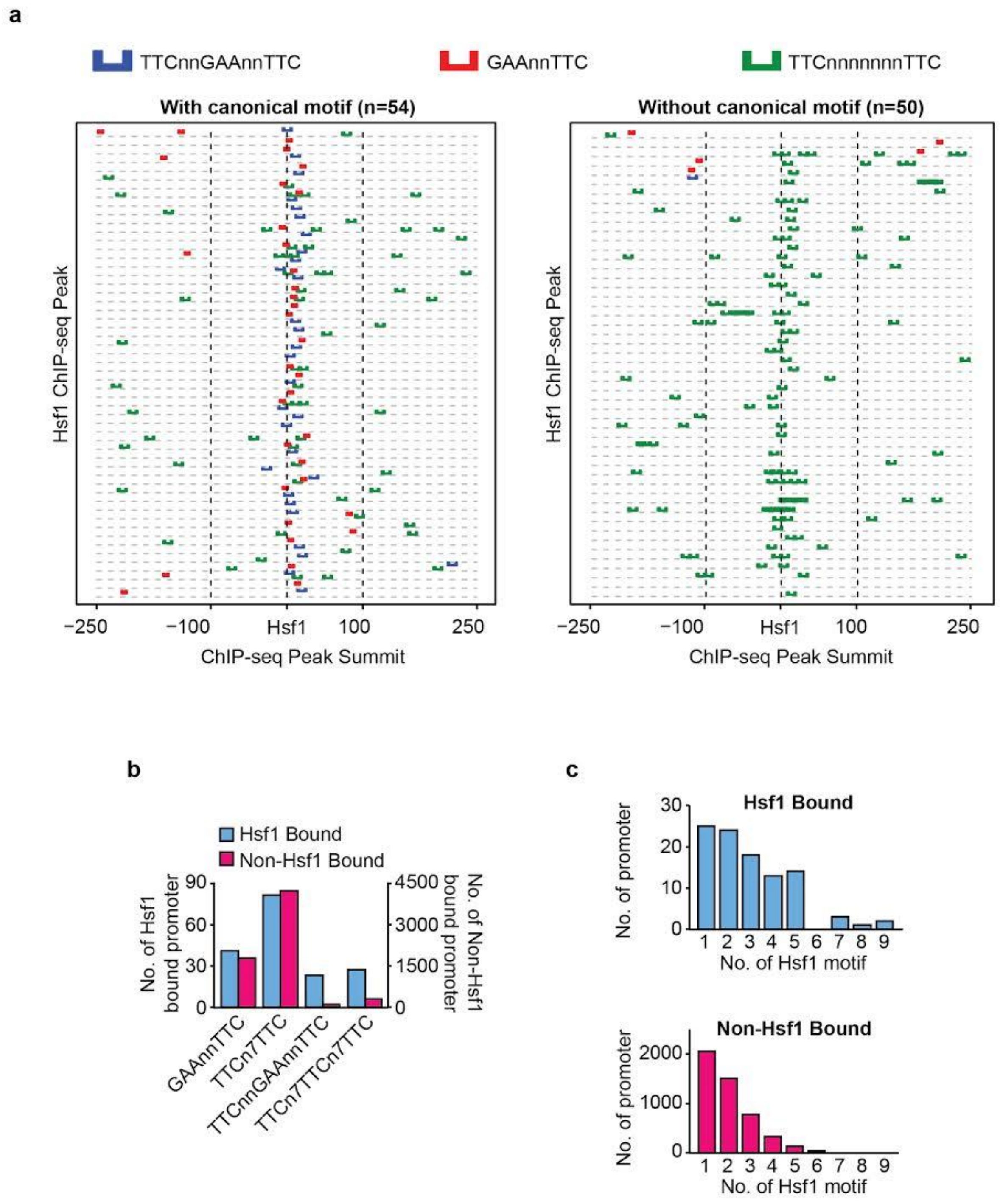

Supplementary Figure 3. Hsf1 binds to multiple related recognition motifs. (a) A schematic illustrating the enrichment of three related motifs (TTCnnGAAnnTTC, GAAnnTTC, TTCnnnnnnnTTC) around the summit of Hsf1-TAP ChIP-seq peaks. Peak lists are separated according to the presence (left) and absence (right) of a previously identified canonical Hsf1 recognition motif (e.g. GAAnnTTC) within 200 bp of the summit. (b) Histogram depicting the number of Hsf1-bound (blue) vs non-Hsf1-bound promoters (red) for each of the motifs identified in our ChIP-seq analysis. (c) Histogram showing the total number of Hsf1 motifs identified in Hsf1-bound (blue, top) and non-Hsf1-bound promoters (red, bottom).

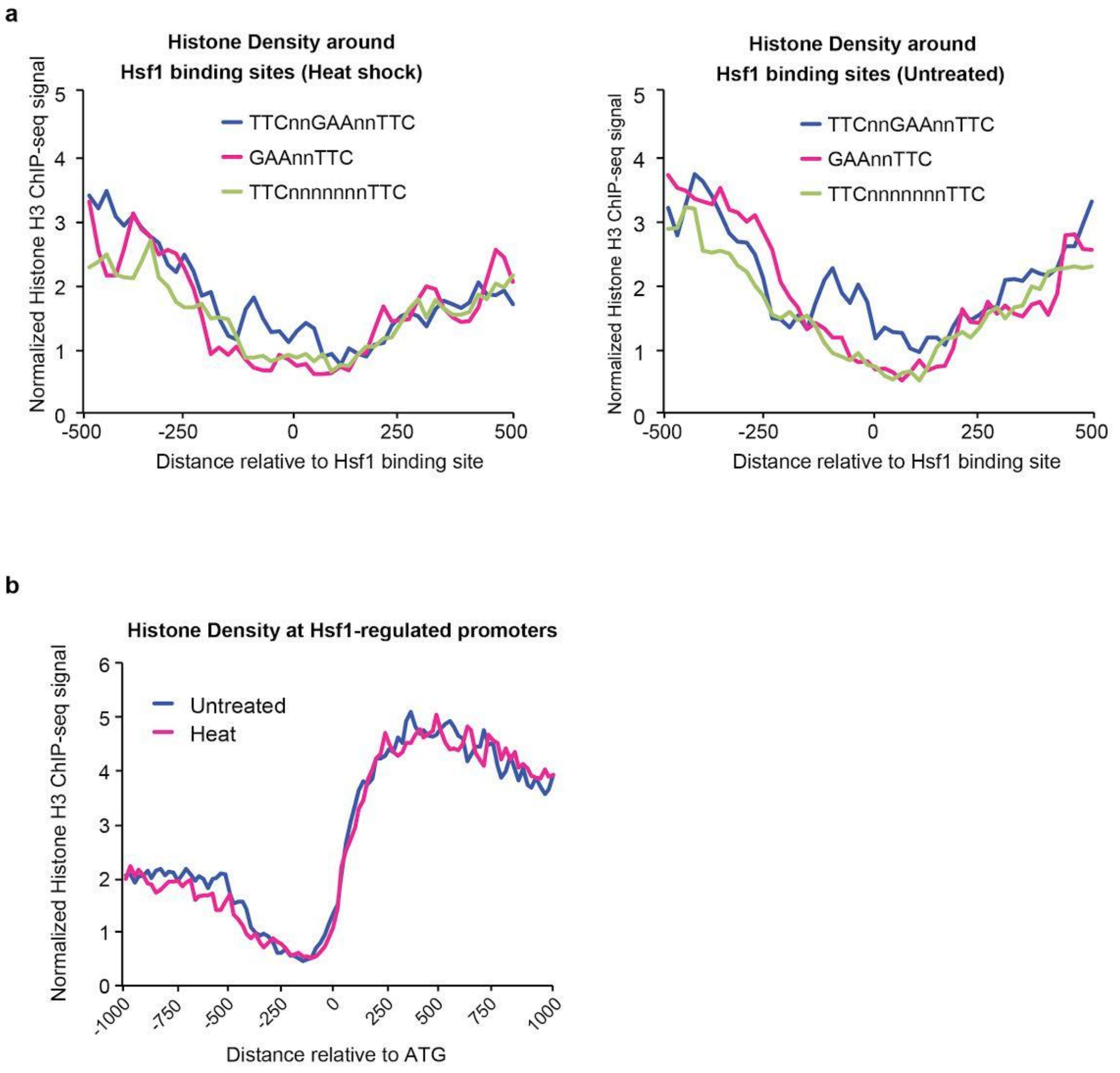

Supplementary Figure 4. Nucleosome density at Hsf1 binding sites and Hsf1-regulated promoters. Line graphs depicting nucleosome levels (a) at Hsf1 binding sites in the presence (heat shock, left) or absence (untreated, right) of a 15 minute 30˚C-42˚C heat shock, containing the TTCnnGAAnnTTC (blue), GAAnnTTC (red) or TTCnnnnnnnTTC (green) Hsf1 recognition motif and (b) at 1kb promoter region of Hsf1 regulated genes, under untreated (blue) and heat shock (red) conditions.

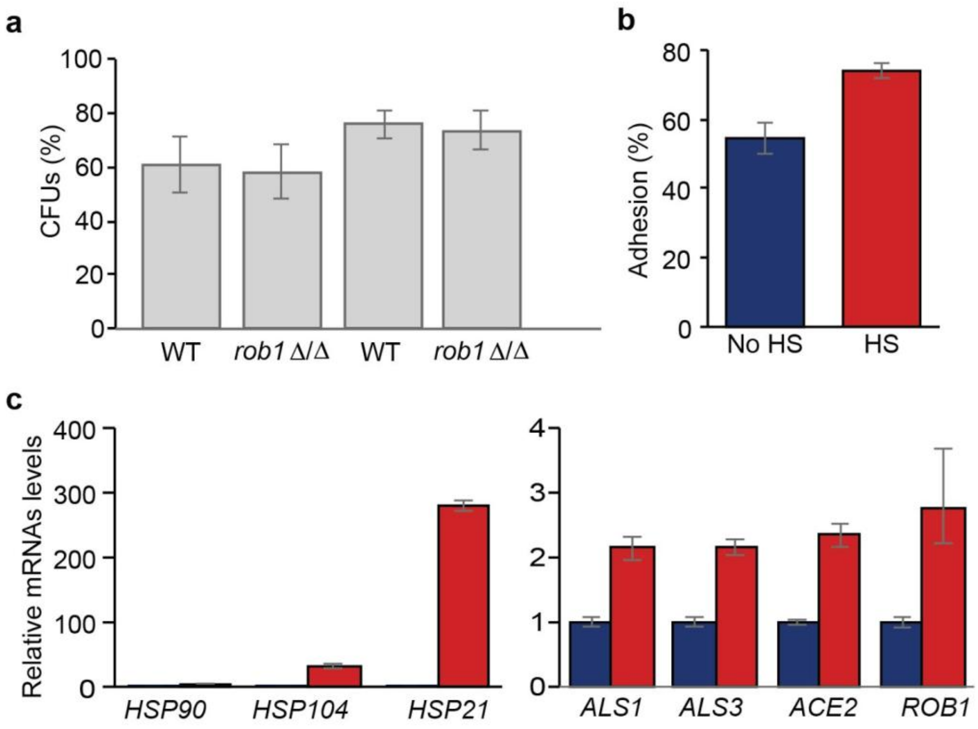

Supplementary Figure 5. Hsf1 targets are induced upon physiologically relevant upshifts but not all targets are required for thermal adaptation. (a) Virulence genes are not required for heat shock survival. Impact of heat shock in deletion mutants of Hsf1 targets and their respective wild-type was determined by measurement of CFUs after a 60 minute 30˚C-42˚C heat shock. % CFUs determined relative to untreated cells. Data represent mean values +/- SEM from three independent biological replicates. Students paired, two-tailed t-test, $p<0.05$. (b) Increased adhesion of *C. albicans* wild-type cells post-heat shock. Wild-type (SN95) cells were grown at 37˚C or subjected to a 15 minute 37˚C to 42˚C heat shock (HS), and 100 cells were added to TR146 monolayers and incubated at 37°C for 90 minutes. After washing, molten Sabouraud's dextrose agar was added and plates were incubated at 30°C for 24 hours. Results expressed as percentage of adhered cells. Data represent mean values +/- SEM. Student t-test, *$P<0.05$ compared to wild type. (c) *C. albicans* wild-type cells were grown at 37˚C or subjected to a 15 minute 37˚C-42˚C heat shock, and transcript levels of target genes were measured and normalised to the *ACT1* loading control. Student t-test, *$p<0.05$ compared to wild type.

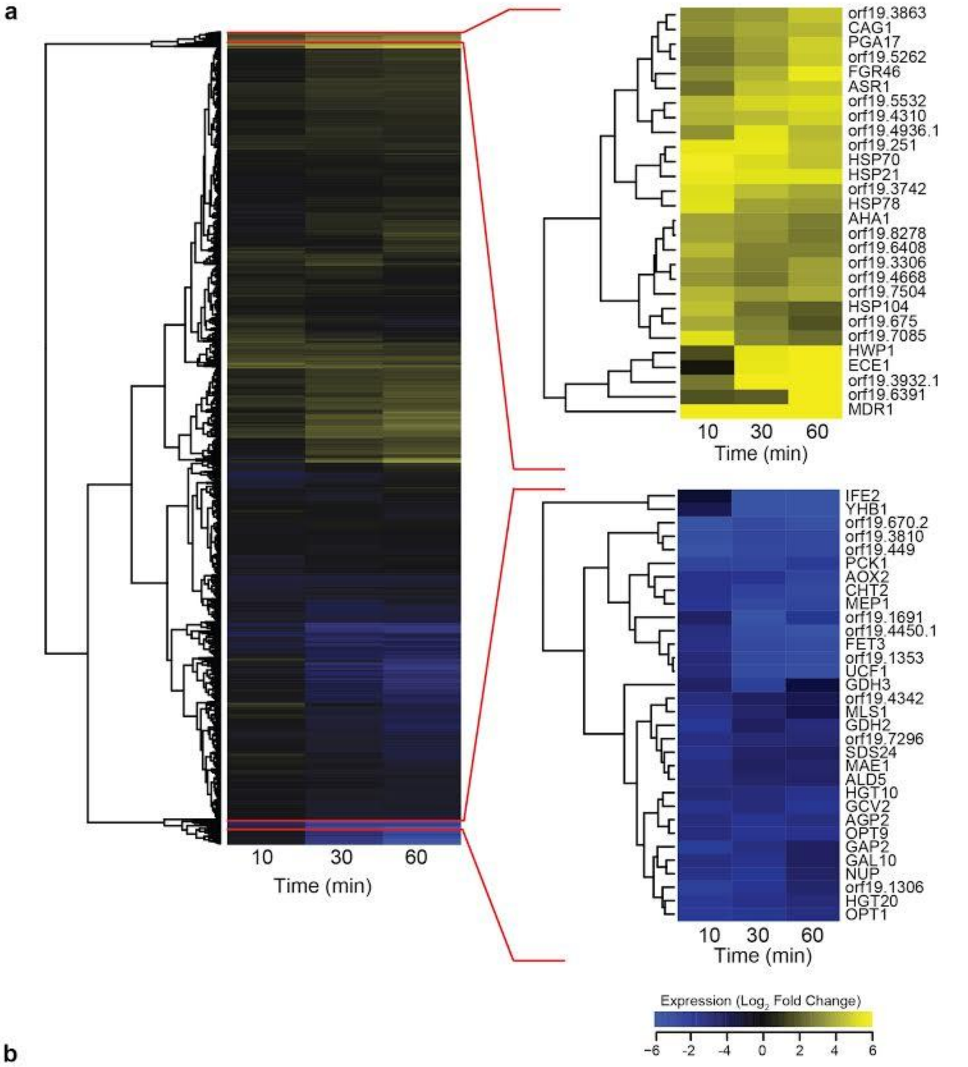

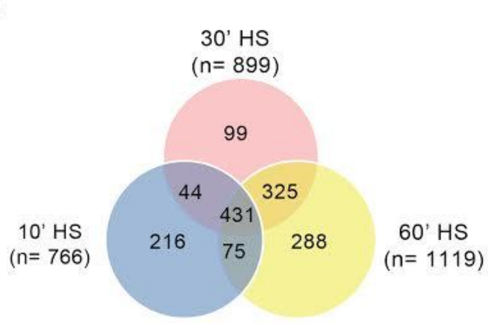

c

GO ID	GO term	*p*-value
6457	Protein folding	1.79E -06
42026	Protein refolding	2.80E -04

Supplementary Figure 6. Global transcriptional response to heat shock. (a) Gene expression pattern of wild-type (SN95) cells 10, 30 and 60 minutes post 30°C-42°C heat shock compared to untreated cells. Genes identified by our analysis were clustered based on their expression patterns. Yellow represents greater than Log_2-fold increase in gene expression, blue represents a Log_2-fold or greater decrease in gene expression. Columns represent individual time points. (b) Venn diagram depicting number of upregulated genes 10, 30 and 60 minutes post 30˚C-42˚C heat shock (HS) in wild-type cells. Overlap between samples is illustrated, with the total number of genes upregulated at each time point represented under each time point. (c) GO term enrichment of genes more than 2-fold upregulated 10 minutes post 30˚C-42˚C heat shock in wild-type cells.

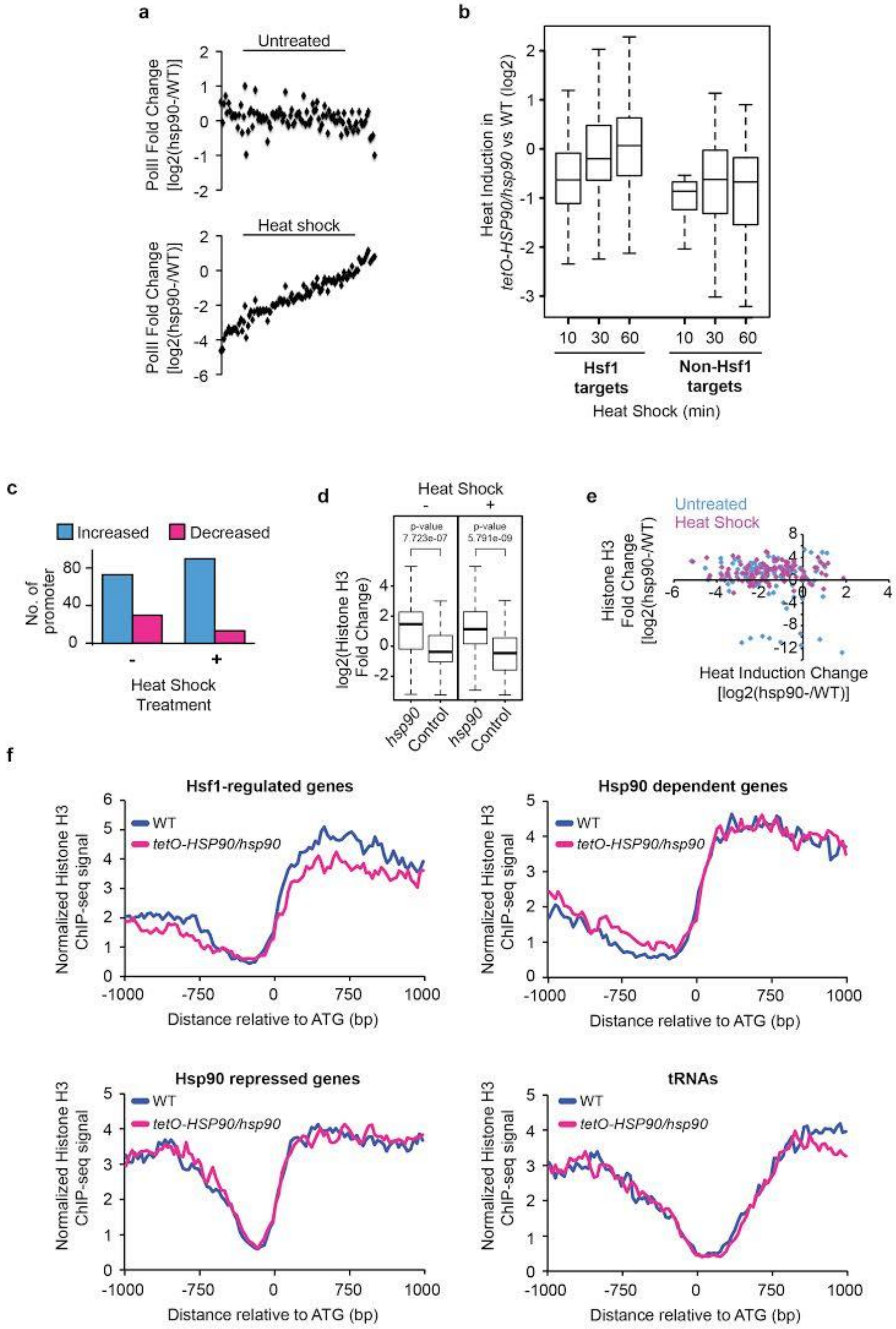

a
Untreated
PolII Fold Change [log2(hsp90-/WT)]
Heat shock
PolII Fold Change [log2(hsp90-/WT)]
b
Heat Induction in tetO-HSP90/hsp90 vs WT (log2)
10 30 60
10 30 60
Hsf1 targets
Non-Hsf1 targets
Heat Shock (min)
c
Increased
Decreased
No. of promoter
Heat Shock Treatment
d
Heat Shock
p-value 7.723e-07
p-value 5.791e-09
log2(Histone H3 Fold Change)
hsp90
Control
hsp90
Control
e
Untreated
Heat Shock
Histone H3 Fold Change [log2(hsp90-/WT)]
Heat Induction Change [log2(hsp90-/WT)]
f
Hsf1-regulated genes
Hsp90 dependent genes
Hsp90 repressed genes
tRNAs
WT
tetO-HSP90/hsp90
Normalized Histone H3 ChIP-seq signal
Distance relative to ATG (bp)

Supplementary Figure 7. *HSP90* depletion delays the Hsf1-mediated heat shock response by increasing nucleosomes at nucleosome free regions of Hsf1- and Hsp90-dependent promoters. (a) Scatter plots illustrating the effect of *HSP90* depletion on transcription of Hsf1 target genes before (untreated) and after heat shock. PolII levels on Hsf1-target genes in the absence of *HSP90* were compared to those in the wild type, and the differences are expressed as $\log_2$ fold-change. (b) Boxplot illustrating expression changes of Hsf1 target genes during heat shock upon depletion of *HSP90* as compared to the wild type (WT) over the indicated time. (c) Histogram depicting the number of Hsf1 target promoters with increased or decreased histone H3 levels upon *HSP90* depletion, compared to the wild type, at their respective nucleosome free regions in both untreated (-) and heat shock (+) conditions. (d) Boxplot depicting the changes in histone H3 levels at the nucleosome free region of Hsf1 target promoters upon *HSP90* depletion, compared to the wild type as in (c) (labelled as hsp90) and between two biological replicates (labelled as Control). The comparisons between biological replicates are to eliminate the possibility that the histone H3 increases are due to a technical artefact instead of a real effect caused by *HSP90* depletion. Two-sample Kolmogorov-Smirnov test was performed between untreated (-) and heat shocked (+) biological replicates. (e) Scatter plot illustrating the correlation between *HSP90* depletion during heat shock for Hsf1 target genes as compared to wild-type (as shown in Figure 5d) and histone H3 levels at nucleosome free regions of the corresponding genes upon *HSP90* depletion under untreated (blue dots) and heat shock (red dots) conditions. (f) Line graphs depicting nucleosome levels between 1kb upstream and downstream of ATG of Hsf1-regulated, Hsp90-dependent, Hsp90-repressed and tRNA genes in wild-type (WT) cells (blue) and *HSP90* depleted (*tetO-HSP90/hsp90*Δ) cells (red).

Supplementary Table 1. ***HSP90*** **depletion drastically changes the global response to heat shock.**

**Wild-type 10 minutes post-heat shock**		
**GO ID**	**GO term**	**P-value**
6457	Protein folding	1.79E-0.6
42026	Protein refolding	0.00028
tetO-HSP90/hsp90 **10 minutes post-heat shock**		
**GO ID**	**GO term**	**P-value**
42026	Protein refolding	1.11E-0.5
6457	Protein folding	0.00016
6458	‘de novo’ protein folding	0.05082
**Wild-type 30 minutes post-heat shock**		
**GO ID**	**GO term**	**P-value**
6457	Protein folding	2.90E-0.7
42026	Protein refolding	0.02073
tetO-HSP90/hsp90 **30 minutes post-heat shock**		
**GO ID**	**GO term**	**P-value**
7017	Microtubule-based process	0.00103
6457	Protein folding	0.00124
226	Microtubule cytoskeleton organization	0.00338
35966	Response to topologically incorrect protein	0.00919
42026	Protein refolding	0.00932
280	Nuclear division	0.06093
22402	Cell cycle process	0.06967
45132	Meiotic chromosome segregation	0.07071
7049	Cell cycle	0.09843
**Wild-type 60 minutes post-heat shock**		
**GO ID**	**GO term**	**P-value**
51087	Chaperone binding	0.02725
51082	Unfolded protein binding	0.5949
tetO-HSP90/hsp90 **60 minutes post-heat shock**		
**GO ID**	**GO term**	**P-value**
280	Nuclear division	0.0043
48285	Organelle fission	0.01157
35966	Response to topologically incorrect protein	0.01495
7059	Chromosome segregation	0.02249
45132	Meiotic chromosome segregation	0.02406
7049	Cell cycle	0.03724
30433	ER-associated ubiquitin-dependent protein catabolic process	0.04685
36503	ERAD pathway	0.04685
7017	Microtubule-based process	0.04937
7067	Mitotic nuclear division	0.05163
22402	Cell cycle process	0.07953
226	Microtubule cytoskeleton organization	0.09115

Genes upregulated more than 2-fold in wild-type and *tetO-HSP90/hsp90Δ* cells were categorised for process enrichment over the 10, 30 and 60 minute 30˚C-42˚C HS.

Supplementary Table 2. *HSP90* depletion induces a heat shock response in the absence of any stress.

**GO ID**	**GO term**	**P-value**
43335	Protein unfolding	0.00163
6979	Response to oxidative stress	0.00233
10286	Heat acclimation	0.00327
70370	Cellular heat acclimation	0.00327
70413	Trehalose metabolism in response to heat stress	0.00327
6081	Cellular aldehyde metabolic process	0.00357
34605	Cellular response to heat	0.00677
46185	Aldehyde catabolic process	0.00814
9408	Response to heat	0.01407
9266	Response to temperature stimulus	0.02277
34599	Cellular response to oxidative stress	0.02427
42026	Protein refolding	0.02961

Genes upregulated more than 1.5-fold in the *tetO-HSP90/hsp90Δ* strain in the absence of any stress were categorised for process enrichment.

Supplementary Table 3. ***C. albicans*** **strains**

**Strain**	**Genotype**	**Source**
SN95	*arg4Δ/arg4Δ his1Δ/his1Δ URA3/ura3:: λimm434 IRO1/iro1::λimm434*	[1]
CaLC2993	*arg4/arg4 his1/his1 URA3/ura3::imm434 IRO1/iro1::imm434, HSF1/HSF1-TAP-ARG4*	[2]
CaLC2928	*arg4/arg4 his1/his1 URA3/ura3::imm434 IRO1/iro1::imm434 TAR-tetO-HSF1::FRT/hsf1*	This study
CaLC1411	*arg4Δ/arg4Δ his1Δ/his1Δ URA3/ura3::λimm434 IRO1/iro1::λimm434 HIS1/his1::tetR-FRT FRT-tetO-HSP90/hsp90::CdHIS1*	[3]
SN152	*arg4/arg4 leu2/leu2 his1/his1 URA3/ura3::immIRO1/iro1::imm434*	[1]
rob1Δ/Δ	*arg4/arg4, leu2/leu2::LEU2(Ca), his1/his1 ::HIS1(Ca), URA3/ura3, IRO1/iro1, rob1/rob1*	[4]
BWP17	*ura3::λimm434/ura3::λimm434, his1::hisG/his1::hisG, arg4::hisG/arg4::hisG*	[5]
als3Δ/Δ	*ura3/ura3::imm434 URA3:: IRO1 als3::ARG4 als3::HIS1 arg4::hisG his1::hisG*	[6]

Supplementary Table 4. Primers for qRT-PCR

Number	Gene	Sequence
oLC2285	CaACT1+855-F	GACCTTGAGATACCCAATTG
oLC2286	CaACT1+1076-R	CAGCTTGAATGGAAACGTAG
oLC2451	CaHsf1+721-F	CCATCAGATGGTATTTCTTCC
oLC2452	CaHsf1+931-R	GGTGAAACACTTGGAAAGTC
oLC756	CaHSP90+1051-F	GCTGAAGAGTTGATTCCAGAAT
oLC757	CaHSP90+1236-R	GGAGAAAGCAGTGTAGAATTGG
oLC1620	CaHSP104+1402-F	CGAGCTAGTCATGAACAATTG
oLC1621	CaHSP104+1660-R	GGATACCAGTCAATCTAGCA
oLC3217	CaHSP21+90-F	CCAAGAAAAATTGCTCAAGG
oLC3218	CaHSP21+325-R	CTTCATCAGGACTCAAATTC
oLC3685	CaALS3 +984 F	GCTACTACCAGAACAGTTAC
oLC3686	CaALS3 +1258 R	GATTAGTATGTGTTGTGGTG
oLC3687	CaALS1 +904 F	GTCTGCAAAGTAAACCTTTC
oLC3688	CaALS1 +1196 R	GTATGATATGGCACATCAAC
oLC2637	CaROB1 +1346F	TGCTGCTTTGATAGCTTCTGG
oLC2638	CaROB1 +1567R	GGAGGGCAATTCCAGATAATTC

SUPPLEMENTARY REFERENCES

1 Noble, S. M. & Johnson, A. D. Strains and strategies for large-scale gene deletion studies of the diploid human fungal pathogen *Candida albicans*. *Eukaryot Cell* **4**, 298-309, doi:4/2/298 [pii] 10.1128/EC.4.2.298-309.2005 (2005).

2 Leach, M. D. & Cowen, L. E. Membrane fluidity and temperature sensing are coupled via circuitry comprised of Ole1, Rsp5, and Hsf1 in *Candida albicans*. *Eukaryotic Cell* **13**, 1077-1084, doi:10.1128/ec.00138-14 (2014).

3 Shapiro, R. S. *et al.* Hsp90 orchestrates temperature-dependent *Candida albicans* morphogenesis via Ras1-PKA signaling. *Curr Biol* **19**, 621-629, doi:S0960-9822(09)00820-3 [pii] 10.1016/j.cub.2009.03.017 (2009).

4 Homann, O. R., Dea, J., Noble, S. M. & Johnson, A. D. A phenotypic profile of the *Candida albicans* regulatory network. *PLoS Genet* **5**, e1000783, doi:10.1371/journal.pgen.1000783 (2009).

5 Wilson, R. B., Davis, D. & Mitchell, A. P. Rapid hypothesis testing with *Candida albicans* through gene disruption with short homology regions. *J Bacteriol* **181**, 1868-1874 (1999).

6 Nobile, C. J. *et al.* Critical role of Bcr1-dependent adhesins in *C. albicans* biofilm formation *in vitro* and *in vivo*. *PLoS Pathog* **2**, e63, doi:06-PLPA-RA-0002R2 [pii] 10.1371/journal.ppat.0020063 (2006).
